# Enhanced Separation Efficiency and Purity of Circulating Tumor Cells Based on the Combined Effects of Double Sheath Fluids and Inertial Focusing

**DOI:** 10.3389/fbioe.2021.750444

**Published:** 2021-10-27

**Authors:** Bo-Wen Li, Kun Wei, Qi-Qi Liu, Xian-Ge Sun, Ning Su, Wen-Man Li, Mei-Yun Shang, Jin-Mi Li, Dan Liao, Jin Li, Wei-Ping Lu, Shao-Li Deng, Qing Huang

**Affiliations:** ^1^ Department of Laboratory Medicine, Daping Hospital, Army Medical University, Chongqing, China; ^2^ Department of Nursing, Children’s Hospital of Chongqing Medical University, Chongqing, China

**Keywords:** circulating tumor cells (CTCs), inertial focusing, particle deflection, double sheath fluids, double spiral microchannel

## Abstract

Circulating tumor cells (CTCs) play a crucial role in solid tumor metastasis, but obtaining high purity and viability CTCs is a challenging task due to their rarity. Although various works using spiral microchannels to isolate CTCs have been reported, the sorting purity of CTCs has not been significantly improved. Herein, we developed a novel double spiral microchannel for efficient separation and enrichment of intact and high-purity CTCs based on the combined effects of two-stage inertial focusing and particle deflection. Particle deflection relies on the second sheath to produce a deflection of the focused sample flow segment at the end of the first-stage microchannel, allowing larger particles to remain focused and entered the second-stage microchannel while smaller particles moved into the first waste channel. The deflection of the focused sample flow segment was visualized. Testing by a binary mixture of 10.4 and 16.5 μm fluorescent microspheres, it showed 16.5 μm with separation efficiency of 98% and purity of 90% under the second sheath flow rate of 700 μl min^−1^. In biological experiments, the average purity of spiked CTCs was 74% at a high throughput of 1.5 × 10^8^ cells min^−1^, and the recovery was more than 91%. Compared to the control group, the viability of separated cells was 99%. Finally, we validated the performance of the double spiral microchannel using clinical cancer blood samples. CTCs with a concentration of 2–28 counts ml^−1^ were separated from all 12 patients’ peripheral blood. Thus, our device could be a robust and label-free liquid biopsy platform in inertial microfluidics for successful application in clinical trials.

## 1 Introduction

Tumor metastasis is the main cause of increased cancer-related mortality (Plaks et al., 2013). Circulating tumor cells (CTCs) carry important information about the primary tumor and thus are crucial in exploring the mechanisms of cancer, metastasis (Esmaeilsabzali et al., 2013), and diagnosis (Chaffer and Weinberg, 2011). The separation of CTCs and subsequent biomolecular analysis can help with the investigation and treatment of cancer (Cohen et al., 2008; de Bono et al., 2008; Alix-Panabières and Pantel, 2013). However, the number of CTCs is rare—only 1–100 per ml of whole blood cells, including billions of red blood cells (RBCs), millions of white blood cells (WBCs), and other background components (Cohen et al., 2008; de Bono et al., 2008; Alix-Panabières and Pantel, 2013). In addition, CTCs will be more vulnerable to attack by immune effector cells leaving the protection of the tumor microenvironment (Mohme et al., 2017). Therefore, rapid isolation of CTCs with high purity and cell activity is a major challenge for CTCs clinical application.

**GRAPHICAL ABSTRACT:**
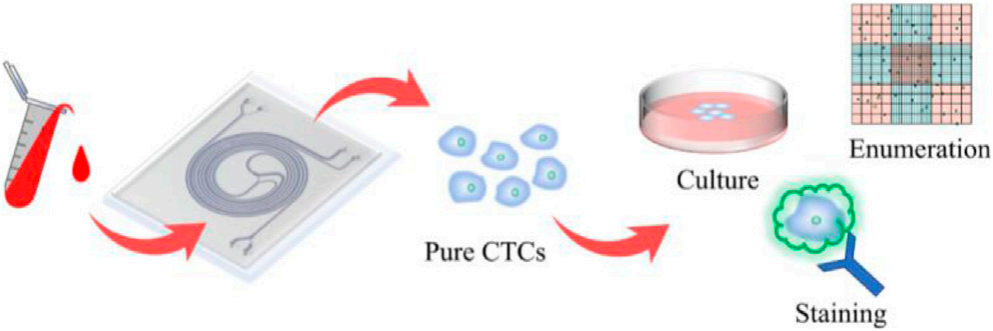
A double spiral microchannel could separate and enrich the high purity and high viability circulating tumor cells from peripheral blood and apply them to downstream analysis, such as culture, staining, and enumeration.

Researchers have found that there are differences in the biological and physical characteristics between CTCs and blood cells (e.g., biomarkers, size, density, and deformability) (Ferreira et al., 2016). Based on these properties, immunomagnetic bead capture (Markou et al., 2011; Chang et al., 2020; Wu et al., 2020), filtering systems (Chang et al., 2015; Yoshino et al., 2016; Xia et al., 2018), and microfluidics (Patil et al., 2015; Abdulla et al., 2020; Zhu et al., 2020) have been developed to isolate CTCs. The CellSearch system, which utilizes immunomagnetic beads coupled with antibodies to identify and isolate CTCs under the effect of external magnetic fields (Riethdorf et al., 2007), has good specificity, and the principle is simple. However, this method is complicated and time-consuming, and there is a possibility of epithelial cell adhesion molecule (EpCAM)-negative CTCs loss. Microfilters have the advantage of simplicity but often ignore CTCs with high deformability and suffer from clogging. In contrast, microfluidics can accomplish sorting using simple device operation with high throughput processing capabilities. With many advantages such as high throughput, low cost, easy operation, and harmless to cells, inertial focusing is one of the most representative and popular techniques in microfluidics (Kuntaegowdanahalli et al., 2009; Xiang et al., 2020; Smith et al., 2021).

Prototypes of inertial microfluidic systems for various applications were discussed by Di Carlo (2009), and he clarified the fundamental fluid dynamic effects. Inertial microfluidic technology applies the effects of the secondary flow of the macroscopic fluid to the microscopic flow channel and thus can separate CTCs from blood cells based on their morphological differences. Hou et al. (2013) developed a classic spiral microchannel with inherent centrifugal forces for continuous, size-based separation CTCs from blood. But the low purity results increased the difficulty of CTC identification and enumeration process. To fully exploit the potential of hydrodynamics separation of inertial microfluidics, it has been proven that designing some special structures in the spiral microchannel, such as micro-obstacles (Shen et al., 2017), micropillars (Zhang et al., 2020), and contraction/expansion microchannel (Gou et al., 2020), can provide stronger Dean effect for efficient and stable particle separation than conventional spiral microchannel. Although each of the above-mentioned sorting methods has its own advantages, when the three important indexes (throughput, purity, and recovery) are increased in parallel, there is a game relationship among them, such as increasing the purity while making a big loss in recovery and increasing the throughput will not meet the high purity. Therefore, in order to overcome this problem, the researchers proposed a multi-stage microfluidic chip combining multiple methods, which made the indexes relatively balanced.

The two-stage sorting proposed by Hou et al. (2013) introduced the tumor cells sorted by the first-stage microchannel into the second-stage microchannel through a catheter to achieve secondary separation and obtained relatively high-purity CTCs. But this chip did not achieve complete integration, and the experimental operation was more complicated. Abdulla et al. (2018) designed an integrated two-stage spiral chip, setting both the primary outlet and secondary inlet on the outer side of the spiral chip, which effectively avoided the use of catheters. In addition, it also further achieved the separation of two types of tumor cells, A549 and MCF-7, with sorting efficiencies of 80.75 and 73.75%, respectively. But, the purity of CTCs was not improved. Similarly, as can be seen in Supplementary Table S2, other studies that used multi-stage microfluidic to separate CTCs suffered from issues associated with either low purity (Sun et al., 2012; Kim et al., 2014; Xiang et al., 2019; Gou et al., 2020). To effectively purify the CTCs, Huang and Xiang (2021) proposed a three-stage i-Mag device combining inertial microfluidics and magnetophoresis for rapid, precise, and tumor antigen-independent separation of rare tumor cells. The purity of the isolated CTC ranged from 50.47 to 93.60%. However, the requirement of a bulk external field generator or complex labeling may limit its practical application. Thus, a simple and fast label-free technique for sorting high purity intact rare cells is capable of being successfully used in clinical practice.

A soft inertial force has been demonstrated in reported studies to quickly and efficiently isolate small cells from larger cells (Wu et al., 2009; Yeh et al., 2017). Using a simple channel geometry with the help of asymmetry sheath flow, a curved and focused sample flow segment would be formed to produce a soft inertial effect on the particles, resulting in large and small particles deflected to different degrees in streamlines and eventually separated. In this study, we proposed a simple yet novel inertial sorting design that incorporates the concept of soft inertial force into a spiral microchannel structure. Soft inertial particle deflection is a simple, size-based method that is advantageous for separating cells or particles of similar size (Wu et al., 2009), but it can be affected by high concentrations of cells/particles, resulting in inaccurate sizes for separation. In contrast, the inertial principle of double spiral microchannel can achieve high throughput cell separation from relatively high concentrations of samples. The first stage of spiral inertial sorting was used to remove the majority of RBCs and WBCs rapidly, and then, the particle deflection effect caused by soft inertial was applied to the removal of smaller cells again, and the second stage of spiral inertial sorting achieved the goal of secondary purification of the CTCs. Experimental results showed that the high separation efficiency, high recovery, high purity, and high viability of CTCs can be achieved from 4% hematocrit (HCT), and the isolated CTCs could be used for further cell culture and enumeration analysis. Thus, this novel double spiral microchannel is a valuable tool for clinical diagnosis and downstream analysis of CTCs.

## 2 Theory and Design Principle

### 2.1 Inertial Focusing of Particles in a Curved Microchannel

The double spiral microchannel was designed as two internally connected single spiral microchannels with inverse directions. The forward and backward flow is described as the 1st and 2nd spiral microchannels, respectively. These were developed to isolate particles based on inertial focusing in a curved microchannel (Rafeie et al., 2019a). When the fluid was flowing through a curved channel due to the centrifugal pressure gradient, two symmetrical vortices were recirculating back to the center along the top and bottom surfaces of the channel. They are called secondary flow or Dean flow (Di Carlo, 2009). Its strength is usually characterized by the dimensionless Dean number:

$$\begin{array}{c} D_{e}=R_{e}\sqrt{D_{h}/2R} \end{array},$$ (1)

where $R_{e}$ is the channel Reynolds number ( $R_{e}=\rho UD_{h}/\mu$ ), ρ is the fluid density, U is the average velocity in the channel, $D_{h}$ is the hydraulic diameter, μ is the dynamic viscosity of the fluid, and *R* is the radius of curvature of the channel. According to Stokes law, the Dean’s drag force on particles suspended in the solution can be defined as:

$$\begin{array}{c} F_{D}=3\pi \mu U_{Dean}a \end{array},$$ (2)

where $U_{Dean}$ is Dean’s flow velocity ( $U_{Dean}=1.8\;\times \;10^{-4}D_{e}^{1.63}$ ), and a is the diameter of the particle. Particles in a microchannel are subjected to an external force called a net inertial lift force ( $F_{L}$ ), which consists of a shear gradient lift force ( $F_{S}$ ) and a wall-induced lift force ( $F_{W}$ ) (Ying and Lin, 2019). The net inertial lift force can be expressed as (Di Carlo, 2009):

$$\begin{array}{c} F_{L}=\;f_{L}\;\left(R_{e},\;x/h\right)\rho U^{2}\alpha^{4}/D_{h}^{2} \end{array},$$ (3)

where $\;f_{L}$ is a non-dimensional lift coefficient that is a function of the Reynolds number and the normalized cross-sectional position (x/h) (Matas et al., 2004).

**FIGURE 1:**
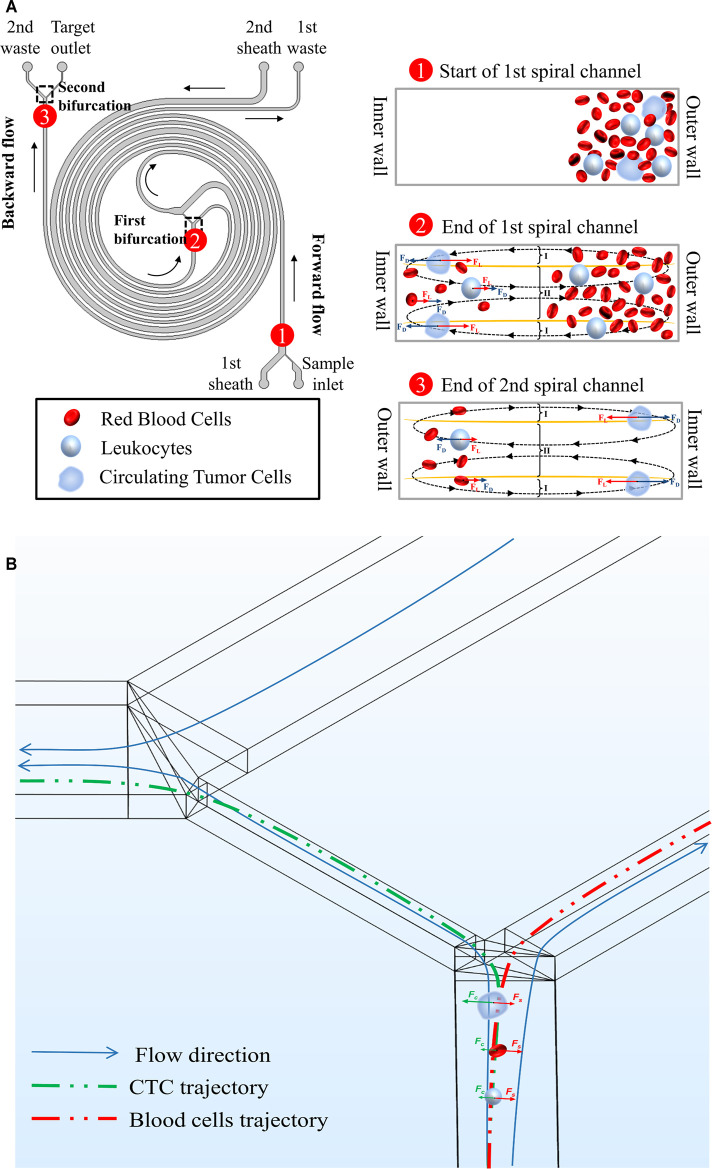
Schematic illustrations and working principles of cell focusing in the double spiral microchannel. **(A)** The working principle of the rectangular cross-section double spiral microchannel to separate CTCs. The defined names of all inlets and outlets were marked in the diagram. Morphologically different cells moved toward different equilibrium positions. Black arrows indicated the flow direction of the fluid. **(B)** Affected by soft inertia, various sized cells were deflected into different outlets at the 1st bifurcation. Green and red arrows showed the migrated direction of CTCs and blood cells.

In our double spiral microchannel, the low diluted blood was directly pumped into the outer wall of the 1st spiral microchannel. The targeted CTCs were finally collected from the inner wall of the 2nd spiral microchannel because of the balance of inertial lift force and Dean drag force while hematologic cells (i.e., RBCs and WBCs) migrated along with the Dean vortices and were eventually recovered from both the 1st and 2nd waste outlets (Figure 1A).

### 2.2 Deflection Characteristics of Particles in the Double Spiral Microchannel

Particles suspended in a fluid with a sufficiently large-particle Reynolds number (*R*_*ep*_) (defined as $R_{e}\alpha^{2}/D_{h}^{2}$ ), such as CTCs, have enough momentum to escape the flow trajectory at the positions of sudden changes in geometric configurations of spiral microchannels (Squires and Quake, 2005). The trajectory mismatch of both particles and fluid could be affected by the Stokes numbers:

$$\begin{array}{c} S_{tk}=\left(\rho_{p}\alpha^{2}U\right)/\left(18D_{h}\mu \right) \end{array},$$ (4)

where $\rho_{p}$ is the particle density. Following Squires and Quake (Squires and Quake, 2005), and assuming the corner radius *R,* the centrifugal force $F_{C}$ acting on the microparticle can be calculated by:

$$\begin{array}{c} Fc=\rho_{p}\pi \alpha^{3}U/6R \end{array},$$ (5)

where $F_{C}$ is equivalent to the $F_{L}$ . And, it is the force that causes the particles to deviate from the initial flow trajectory. The deflection will be balanced by the Stokes drag, which can be estimated by:

$$\begin{array}{c} F_{S}=3\pi \mu \alpha U_{m} \end{array},$$ (6)

where $U_{m}$ is the particle migration velocity in the fluid. The $F_{S}$ is equivalent to the $F_{D}$ . The $F_{D}$ and $F_{S}$ are proportional to $\alpha^{3}$ and α *,* respectively. The CTCs are dominated by $F_{C}$ to deflect into the connection channel between the 1st and 2nd spiral microchannels while the blood cells are subject to Stokes drag to follow the main fluid streamlines and migrate into microchannel connected to the 1st waste (Figure 1B).

### 2.3 Design Principle of Double Spiral Microchannel

The double spiral microchannel is different from the typical cascade single spiral microchannels (Hou et al., 2013; Abdulla et al., 2018; Kim et al., 2014; Robinson et al., 2017). Our design was directly constructed using two spiral microchannels in a single microfluidic chip (Supplementary Figure S1), which reduced the cost of manufacturing and the complexity of experimental operations. Although the sheath-less technologies were regarded as methods simple and easy to control (Chung et al., 2013; Huang et al., 2016; Shen et al., 2017; Gou et al., 2020), the double spiral microchannel had the conceptual advantage of a simple structure that was significantly more accessible and available to the non-specialist, and the shorter channel length greatly reduced the resistance within the microchannel, which was beneficial for cellular activity. In addition, all inlets and outlets were extended to the outer edges of the microchannel, which facilitated the connection between the chip and the tubing and obtained a better viewing field during the experiment (Supplementary Figure S2).

The major microchannel had a 3.5-loop Archimedes spiral structure with a rectangular cross-section having a low aspect ratio (∼1:3; h: 170 μm; w: 500 μm). In the low-aspect-ratio rectangular spiral microchannel, the particles are transferred along the longer cross-section toward two central equilibrium positions which implies the number of particle equilibrium positions can be reduced (Zhang et al., 2016), which are widely used as highly efficient cell-sorters (Nivedita et al., 2017). The design of the microchannel height followed the inertial focus criterion: α/H>0.07 (Bhagat et al., 2009), which allowed the inertial focusing of only large target cells near the inner wall. In order to keep the smaller untargeted cells far away from the inner wall, the sheath fluids were introduced so that all the cells entering the spiral channel and beginning their lateral migration at a similar position. And the length of the microchannel was designed to ensure that small particles can accomplish a complete 1 Dean cycle (DC 1) (Hou et al., 2013). A gap of 500 μm was reserved between the flow channel to strengthen pressure without leakage. We specially designed the microchannel connected with the 1st waste to balance the bifurcation pressure between the first and second spiral microchannels, which followed the concept of the cascade spiral system (Hou et al., 2013; Abdulla et al., 2018). The areas where the sample and sheath fluid converged were designed as pear-shaped transitions, which increased the stability of the convergent flow. In addition, the initial radius of the 2nd microchannel was set to 0.4 cm in order to ensure that the connecting channel between the two-stage microchannel had sufficient length. Detailed dimensions of the double spiral microchannel are provided in the Supplementary Figure S10 and Supplementary Table S1.

Versus traditional single spiral microchannel, our double spiral microchannel could integrate the first-stage forward flow (1st spiral microchannel) and second-stage backward flow (2nd spiral microchannel). As reported in the similar curved microchannel (Rafeie et al., 2019a; Rafeie et al., 2019b), yellow curves represent where the magnitude of the x-component Dean velocity is zero, and the cross-section can be split into three regions (Figure 1A). At the end of the forward flow microchannel, the Dean flow was stronger at I regions, while at the end of the backward flow microchannel, the Dean flow was stronger in the region II. This prompted larger particles/cells focused on the inner wall of the microchannel in the forward and backward flow microchannels, and smaller particles/cells were dominated by Dean drag force and moved back and forth regularly between the inner and outer walls of the double spiral microchannel. When the smaller particles/cells returned back to the outer wall, they completed DC 1 (Supplementary Figure S3). However, they were not in equilibrium, and the number of particles was relatively large, so a portion of particles could not complete DC 1. And the existence of this part of the particles might compromise the separation efficiency and purity of the large particles. Coincidentally, the 2nd sheath increased the resistance to flow at the end of the forward flow microchannel, which caused most of the fluid to be deflected into the microchannel connected to the 1st waste outlet. Depending on the deflection characteristics of the different particles, larger particles (i.e., CTCs) and smaller particles (i.e., WBCs and RBCs) entered the backward flow microchannel and the 1st waste channel, respectively. Fewer small particles in the backward flow microchannel would discard from the 2nd waste. Therefore, the combination of double sheath fluids and inertial focusing could efficiently isolate CTC and enhance its purity.

## 3 Materials and Methods

### 3.1 Chip Fabrication

Both single and double spiral microchannels (Supplementary Figure S1) were fabricated using a standard soft lithography process according to standard protocols (Hanguang Co., China). The device was made by casting degassed polydimethylsiloxane (PDMS) in 10:1 ratio with a curving agent (Sylgard 184, Dow Corning Inc.) on the mold and subsequently baking it in an oven at 80°C for 1 h. After curing, the PDMS slab was peeled off from the master mold and punched through at the inlet and outlets with a simple puncher (Hanguang, China). The PDMS was bound to a glass substrate treated with a plasma cleaner (PDC-002, Harrick Plasma) for 1 min. Finally, the completed device was placed in an oven at 80°C for 3–4 h to enhance the bonding performance.

### 3.2 COMSOL Modeling

The numerical calculation of the double spiral microchannel was completed by the finite element analysis software COMSOL Multiphysics (COMSOL Inc.). Modeled the three-dimensional of the double spiral microchannel, the visualization and analysis of the microfluid were achieved by combining simulation analysis and data computation processing, which referenced the published literature (Hou et al., 2013). The model was meshed using a refined physical field control grid. Numerical simulations were performed in the computational domain using incompressible Newtonian laminar flow to explore the interaction among the different inlet flows at appropriate initial conditions (specific laminar flow rate for inlet and zero pressure for outlet) and slip-free wall boundary conditions. The computational domain was calculated by solving the non-simplifying Navier-Stokes equations, and its nonlinear inertia terms were not negligible. Although the trajectories of the particles were not tracked, we summarized the potential force action on the particles depending upon the numerically flow field and the basic theory and predicted the potential particles’ trajectories.

### 3.3 Sample Preparation

#### 3.3.1 Fluorescent Polystyrene Microspheres

Two types fluorescent polystyrene microspheres (Spherotech, United States) with diameters of 10.4 μm (10^5^ cells ml^−1^) and 16.5 μm (10^3^ cells ml^−1^) were selected to mimic the behaviors of WBCs and CTCs, respectively. The microsphere solutions were diluted with phosphate-buffered saline (PBS; HyClone, United States); 0.1% v/v Tween-20 (SIGMA, United States) was prevented the particles from settling prematurely; 0.5% v/v BSA (SIGMA, United States) was added to avoid non-specific adhesion of the particles in the microchannel walls.

#### 3.3.2 Cancer Cell Lines

Three types of human cancer cell lines (i.e., MCF-7, Hela, and K562) were cultured according to the manufacturer’s instructions. The pellets of cultured cell lines were prepared by centrifugation at 300 g for 5 min and then resuspended in the medium without fetal bovine serum (FBS; HyClone, United States). Finally, they were stained with Cell Tracker Orange CMTMR Dye (Invitrogen, United States) for 30–45 min according to the manufacturer’s instructions. A known number (10^2^–10^3^ cells ml^−1^) of tumor cells were then spiked into healthy peripheral blood to mimic actual clinical samples. The number of fluorescently stained cell lines was determined using a blood cell counting plate (Qiujing, China) in triplicate experiments.

#### 3.3.3 Clinical Samples

The peripheral blood from healthy volunteers and carcinoma patients were collected in vacutainer tubes coated with EDTA-K2 anticoagulant. The ethics approval was obtained from our Medical Institutional Review Board (Approval number: 2020P27). Before experiments, the whole blood was diluted with 0.9% saline or treated with RBC lysis buffer (Thermo Fish Scientific, United States) according to the manufacturer’s instructions. All blood samples were used within 24 h.

### 3.4 Immunofluorescence Staining

The cell suspension was dropped onto the poly-lysine-coated cell climbing cover glass for overnight incubation. According to the cell status, the cover glasses were taken out at the right time for 24 h or more. For suspension cells, they have used suspension immunofluorescence staining. The staining step followed a recommended protocol. In brief, cells were incubated with CD45 firstly (Milteny Biotech, Germany). After washing with PBS, they were fixed with 4% formaldehyde for 30 min. The cells were then incubated with cytokeratin antibody (Milteny Biotech, Germany). Finally, cell nuclei were stained with DAPI (Beyotime, China) for 5–15 min. The slides were then observed with an inverted fluorescence microscope.

### 3.5 Cell Viability Assay

We performed short-term (0 h) and long-term (24 and 48 h) viability experiments on cells collected from the target outlet. The PE Annexin V Apoptosis Kit I (BD Biosciences, United States) was used to detect short-term cell viability. The kit contains two dyes, PE Annexin V and 7-Amino-Actinomycin (7-AAD), which can detect early apoptotic cells (PE Annexin positive, 7-AAD negative), late apoptotic cells or dead cells (PE Annexin positive and 7-AAD positive), and viable cells (PE Annexin and 7-AAD negative). Cell viability was calculated as the ratio of the number of viable cells to the total number of cells. For the long-term cell viability, the collected cells were cultured in 24 wells (Corning, United States) for further observation of the cell viability and morphology after going through the chip at 24 and 48 h. All cell viability tests were designed with the control groups.

### 3.6 Experimental Setup and Data Analysis

The double spiral microchannel was mounted on the carrier stage of an inverted microscope (Olympus IX73, Japan). The syringe pumps (LongerPump, China) and connection 1/16″ FEP Tubing (CorSolutions, United States) were used to provide different sample injection rates (Supplementary Figure S2). A magnetic stirrer (V&P Scientific Inc., United States) ensured uniform distribution of particles/cells in the sample. In bright-field mode, the particle/cell dynamics over a certain period of time were continuously captured using a high-speed camera (FASTCAM NOVA S9, United States) and saved in video format.

We opened the video *via* ImageJ software (NIH) and performed the “Image-Stacks-Z Project” operation. Each frame was then superimposed to characterize cell/particle movement in the video *via* the Z direction to get a clear cell/particle trajectory map for measurement and analysis. Concentrations of initial samples and collection from different exit samples were determined using a blood cell counting plate to characterize the sorting and enrichment performance. Detailed calculation formulas are displayed in the Supplementary Document S1. All statistical analyses were performed using GraphPad Prism (GraphPad Software Inc., United States). Data are presented as means ±SD. An unpaired *t*-test was used to analyze the difference between the two groups. *p* < 0.05 *via* a two-tailed test was considered significant.

## 4 Results and Discussion

### 4.1 Simulation of Velocity Distribution and Understanding Physics

**FIGURE 2:**
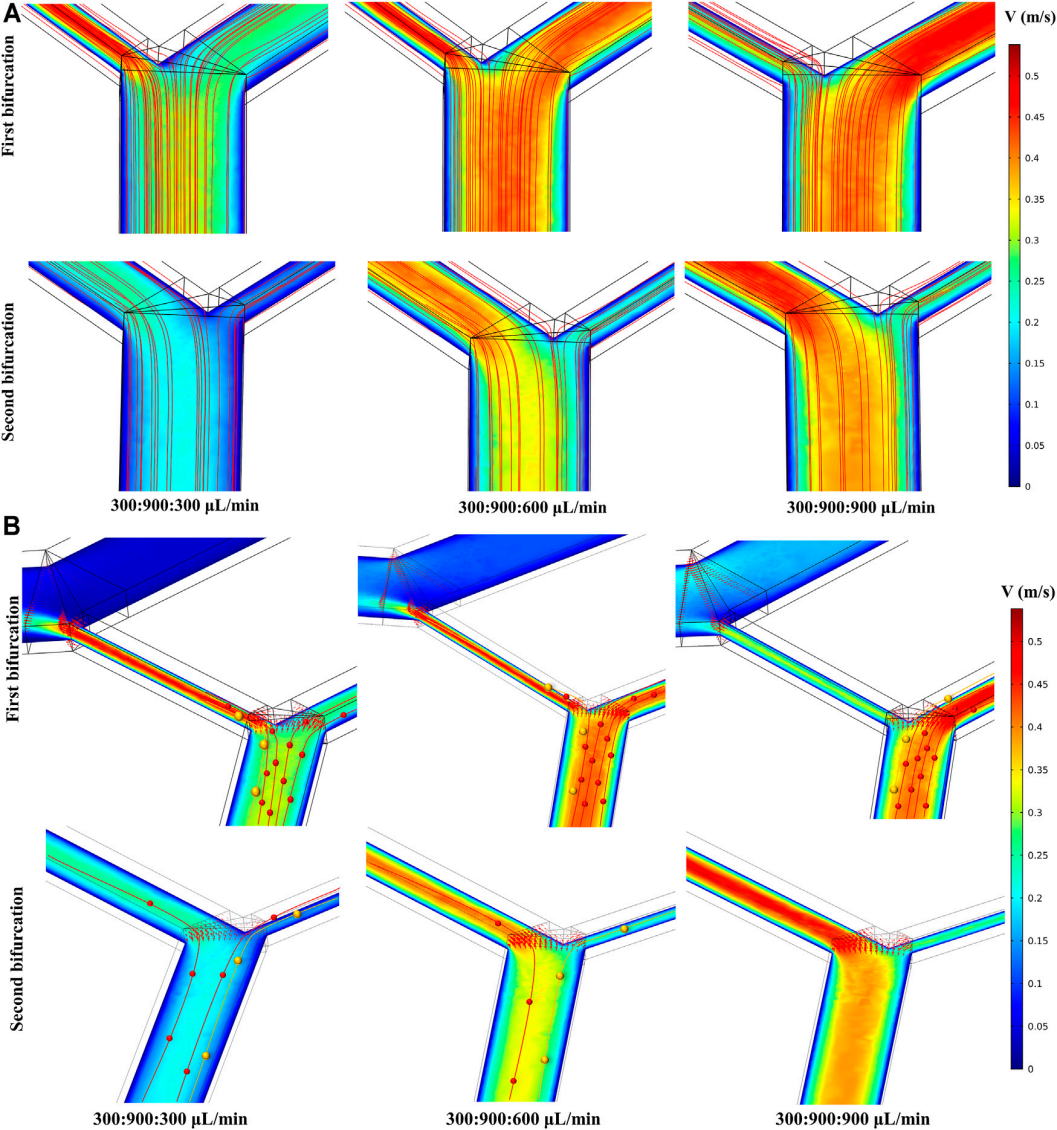
Simulation results at two bifurcations. The flow ratio at the bottom of the image corresponds to sample:1st sheath:2nd sheath. **(A)** The influence of the 2nd sheath flow rate on the both velocity and fluid streamline distribution at the two bifurcations. **(B)** Particle trajectories were drawn on the simulation of velocity distribution. Different sized particles moved toward different outlets.

The COMSOL CFD model was used to simulate the velocity and streamline distribution in the double spiral microchannel to guide the experimental conditions (Figure 2 and Supplementary Figure S4). Ensuring comparable particle separation in the 1st and 2nd microchannels required consistent fluid states in the two-stage microchannel, in which the 2nd sheath played a key role. By adjusting the flow rate of the second sheath to compare and analyze the flow rate in different inlet and outlet cross-sections, the flow rate distributions of the two bifurcations were similar when the flow rate of the 1st sheath was equal to that of the 2nd sheath (Supplementary Figures S4A,B). The microchannel connecting the 1st waste was the crucial balance channel of the chip, and its width was set to 350–395 μm. When the flow rate of the 2nd sheath varied from 300 to 900 μl min^−1^, the pressure distribution showed a gradual drop from the inlets to the outlets, indicating that the pressure in the two-stage microchannel was relatively balanced and the fluid could flow steadily (Supplementary Figure S4c). Since the bifurcation directly reflected the efficiency of particle/cell separation, we analyzed the fluid state in the vicinity of the two bifurcations and obtained the horizontal section velocity and streamline distribution (Figure 2A). The results showed that the magnitude of the 2nd sheath was a direct factor affecting the flow rate at the bifurcation. As its flow rate increased, the more fluid streamlines would flow into the 1st waste channel.

Considering inertial focusing, particle deflection theory (Wu et al., 2009; Squires and Quake, 2005), and the analysis of the simulation results of the flow velocity and fluid streamline distribution, we summarized the potential particle trajectory in the double spiral microchannel as follows. The particle streams that had completed inertial sorting would be influenced again by the flow velocity distribution when it passed through the bifurcation. As shown in Figure 2B, when the flow rate of sample, 1st sheath, and 2nd sheath was adjusted to 300, 900, and 900 μl min^−1^, respectively, the Stokes resistance of both large yellow and small red particles rose, resulting in yellow and red particles at the first bifurcation following the deflected trajectory of the fluid into the microchannel connected to the 1st waste. Few or no particles entered the 2nd spiral microchannel, and thus, no particles appeared at the 2nd bifurcation. However, keeping the flow rate of the sample and the 1st sheath constant and adjusting the flow rate of the 2nd sheath to 300 μl min^−1^, the velocity distribution within the two-stage microchannels became inconsistent (Supplementary Figure S4B). In addition, the intermediate channel connecting the two-stage microchannel had less resistance than the 1st waste channel owing to the lower flow rate in the 2nd microchannel. This unbalanced pressure drove more small particles with a fluid deflection into the 2nd spiral microchannel and eventually collected from the target outlet. Adjusting the flow rate of the 2nd sheath to 600 μl min^−1^, the velocity distributions in the first- and second-stage microchannels were close to each other, which ensured that the particle motion in the two microchannels remained basically the same. Furthermore, a part of the streamline trajectories at the 1st bifurcation were deflected into the 1st waste channel, and particles of different sizes were subject to different degrees of deflection at the end of inertial sorting to achieve secondary separation. Compared with the cascaded spiral microchannels detailed in previous studies (Hou et al., 2013; Abdulla et al., 2018), our uniquely designed double spiral microchannel can serve as forward and backward two-stage spiral separation with the introduction of particle deflection method at the 1st bifurcation. This two-stage inertial separation combining two sorting methods can effectively remove more small particles, further improving the separation efficiency and the purity of the large target particles.

### 4.2 Parameter Optimization and Comparative Analysis Using Binary Fluorescent Polystyrene Microspheres

To visualize and analyze the different particle trajectories, fluorescent microspheres of 16.5 and 10.4 µm were labeled in yellow and red, respectively, and the experimental conditions were further optimized depending on the sorting process and results. The 16.5 μm fluorescent microspheres satisfied α/H>0.07 , allowing fully inertial focusing within the microchannel. Their inertial lift force was close to the magnitude of the Dean drag force, so there existed the equilibrium position within the microchannel. However, the 10.4 μm fluorescent microspheres were subjected to Dean drag force greater than the inertial force, so they followed the Dean vortex to do periodic cyclic.

**FIGURE 3:**
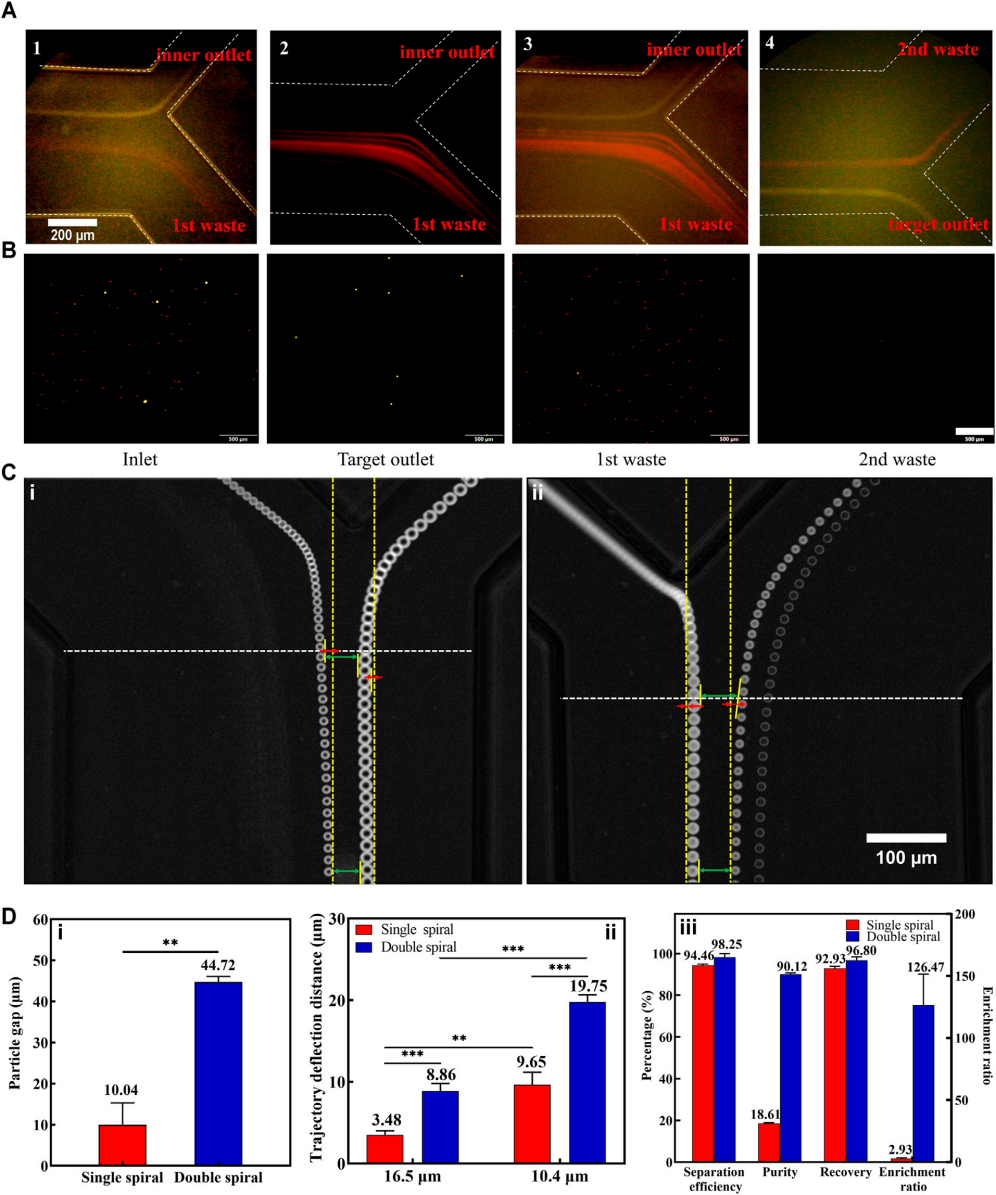
Results of fluorescent microspheres for separation. **(A)** The captured fluorescent trajectories of 16.5 μm (1) and 10.4 μm (2) at the first bifurcation. Composite images at the first (3) and second (4) bifurcation. The white dotted lines indicate the microchannel walls. **(B)** Microspheres (16.5 μm with yellow fluorescence and 10.4 μm with red fluorescence) collected at the inlet and different outlets. The scale bar is 500 μm. **(C)** The representative superimposed images of 16.5 and 10.4 μm microspheres at the first bifurcation of both single **(i)** and double **(ii)** spiral microchannels. Some measurement reference lines were marked in the panel. **(D) (i)**: The particle gaps between 16.5 and 10.4 μm microspheres in single and double spiral microchannels. **(ii)**: The particle trajectory deflection distances in single and double spiral microchannels. **(iii)**: Comparative results of methodological parameters between single and double spiral microchannels. **p < 0.05, ***p < 0.001.

First, we optimized the experimental parameters of the first-stage microchannel. When the total flow rate (sample and 1st sheath) ranged from 700 to 1,300 μl min^−1^, the 16.5 μm microspheres were focused near the inner wall of the microchannel, while the 10.4 μm microspheres underwent a migration movement from the inner wall to the outer wall (Supplementary Figure S5A). Determining that the optimal total flow rate for separating the two kinds of microspheres was 1,200 μl min^−1^, we then explored the effect of the sample-to-sheath flow rate ratio on microsphere sorting. As the flow rate of the sample rose gradually, the particle streamlines width of the two types of microspheres widened (Supplementary Figure S5b), which increased the risk of target microspheres loss. This phenomenon arose due to a great number of particles passing through the cross-section in unit time, resulting in an increase in the interaction forces between the particles (Warkiani et al., 2016). Consequently, weighed between the sorting throughput and efficiency, the optimal flow rate ratio of sample to 1st sheath was 1:3 (300:900 μl min^−1^, Figure 3A).

A small amount of 10.4 µm microspheres still entered the second-stage microchannel during the experiment (Supplementary Figures S5C,D). And a small part of these microspheres entered the target outlet. There were two reasons for this phenomenon: one was that the 10.4 µm microspheres’ particle streamline was too wide and part of the 10.4 µm microspheres overlapped with the 16.5 µm microspheres’ position; the other reason was that the 16.5 µm microspheres migrated to the inner wall together with some of the 10.4 µm microspheres as they focused near the inner wall, or the 10.4 µm microspheres were blocked by the 16.5 µm microspheres as they migrated from the inner wall to the outer wall. The above phenomenon could be effectively avoided by reducing the concentration of microspheres. However, the cell concentration in the actual sample is higher, so it is necessary to improve the separation efficiency of 16.5 µm microspheres by optimizing the flow rate of the 2nd sheath. Continuously increasing the flow rate of the 2nd sheath, it was found that fewer and fewer 10.4 µm microspheres entered the second-stage microchannel. Subsequently, a high-speed camera was used to precisely capture the microspheres’ trajectories and measure the change in particle-free gap (the difference between the lengths of upper and lower green arrows in Figure 3C) and the particle deflection distance (the length of the red line in Figure 3C). The yellow dotted line parallel to the sidewall of the microchannel was considered as the initial trajectory position of the particles, and it was used as a reference line to quantify the degree of particle deflection. It turned out that the 2nd sheath introduced the particle deflection effect at the 1st bifurcation, which was consistent with the simulation results.

To further demonstrate the unique particle deflection effect in the double spiral microchannel, we performed comparative experiments between single and double spiral microchannels. The results showed that the deflection effect in the double spiral microchannel was four times greater than that in the single spiral microchannel, and the gap distance between the microspheres was significantly amplified (Figure 3D). In addition, the deflection trajectories of 16.5 and 10.4 µm microspheres in the double spiral microchannel were significantly larger than those in the single spiral microchannel, which also explained that the 16.5 µm microspheres could eventually enter the second-stage spiral microchannel although they exceeded the critical line of the 1st bifurcation. The flow rate of the 2nd sheath was finally set to 700 μl min^−1^. At the same time, it also met the velocity requirement for inertial focusing of the 16.5 μm microspheres in the 2nd spiral microchannel (Supplementary Video S1). This design of the double spiral microchannel not only amplified the size difference in particle trajectories caused by lateral inertial deflection and secondary flow but also reduced the possibility of residual small particles entering the target outlet (Figure 3B). The particle deflection effect played a vital role in manipulating small particles (Squires and Quake, 2005), effectively enhancing the separation efficiency and purity of 16.5 μm microspheres. The results showed that 97% of the 16.5 μm fluorescent microspheres were collected from the target outlet with 98% separation efficiency, 90% separation purity, and 126-fold enrichment rate. These data indicate that the double spiral microchannel has the potential to be a powerful tool for separating rare cells.

### 4.3 Isolation of Spiked Tumor Cell Lines from Blood Samples

There are many cells in the peripheral blood, which will influence the focused migration movement of tumor cells within the microchannel. To reduce the number of background cells, we chose RBC lysis or dilution methods for whole blood. By comparing the two pretreatment methods, RBC lysis required additional centrifugation steps that were time-consuming and decreasing the number and cell viability of CTCs (Supplementary Figure S6), which demonstrated that the dilution method could minimize their unnecessary loss. Therefore, we optimized the dilution factor of whole blood. Peripheral blood was diluted to different HCT values of 4.0, 2.0, 1.0, and 0.8%. At the end of the 1st spiral, we measured and analyzed the width of the cell distribution at different dilution factors (Supplementary Figure S7). The width of the cell distribution gradually narrowed with the increase of the dilution and the 2nd sheath flow rate. At a higher flow rate of the 2nd sheath, the cell distribution width of 4.0% HCT was consistent with that of 1.0% HCT under a lower 2nd sheath, which proved that the effect of high 2nd sheath flow in reducing the cell distribution width was greater than the dilution. Enumerated the blood cells from different outlets, it was found that 99.999% of blood cells could be removed after performing the two-stage separation. Compared with the recently passive microfluidic devices (removal rate of blood cells <99.96%) (Huang et al., 2016; Xiang et al., 2019), approximately another 10^5^–10^6^ blood cells per ml of recovered fluid were reduced (Supplementary Figure S8A). In addition, we performed the secondary separation of the single spiral microchannel for comparison with the double spiral microchannel. The results showed there were fewer blood cells at the target outlet in the double spiral microchannel. The separation effect of the double spiral microchannel was superior to the secondary separation effect of the single spiral microchannel (Supplementary Figure S8B).

**FIGURE 4:**
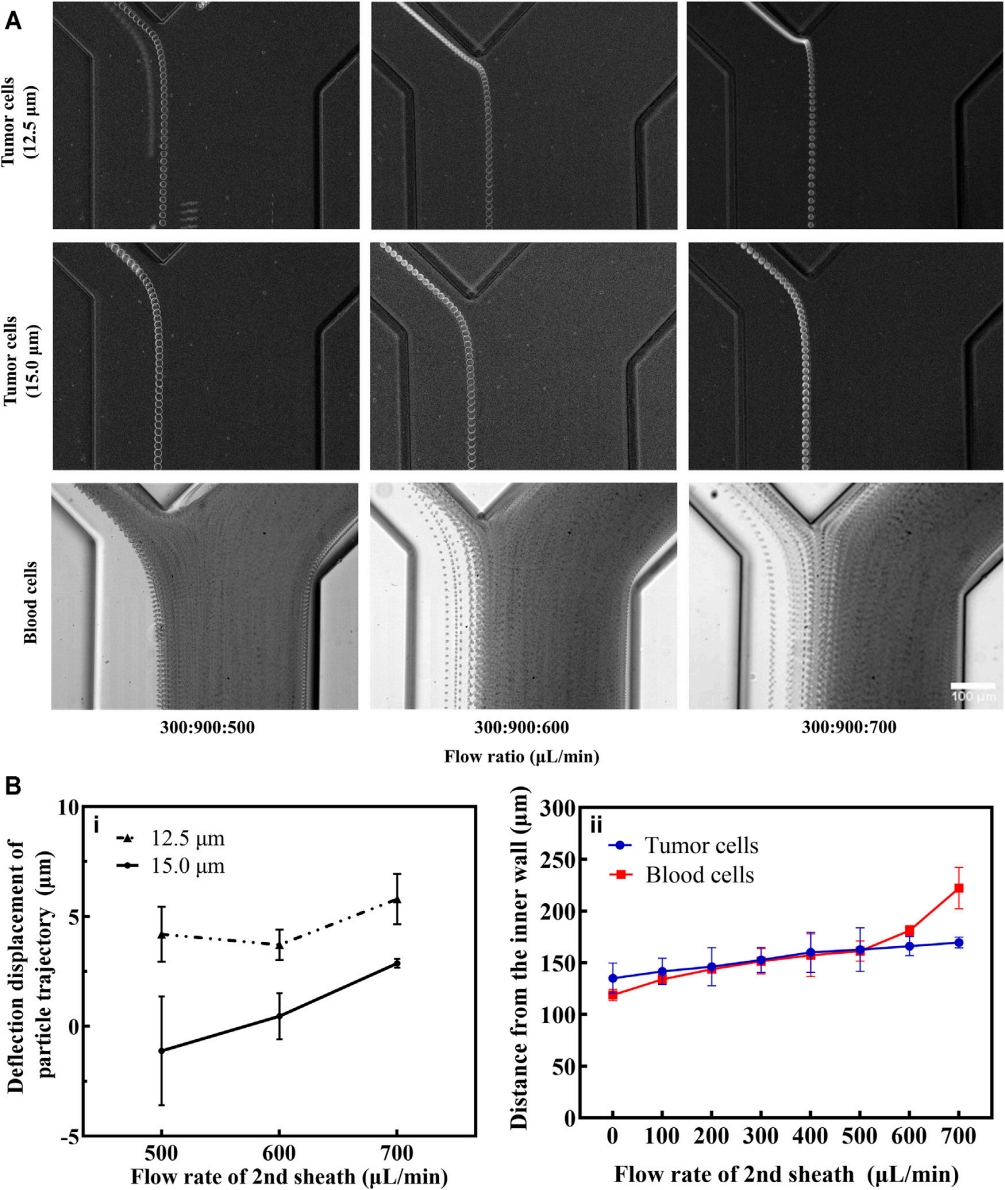
Measurement and analysis of particle trajectories of CTCs and blood cells. **(A)** Tumor cells and blood cells movement trajectories under different flow rates for the 2nd sheath (ImageJ stacks processing). The scale bar is 100 μm. **(B) (i)**: considering the bisector at the bifurcation as the reference line, the deflection displacement of different tumor cells was quantitatively measured (a value greater than zero means that cells are deflected in the direction of the 1st waste and vice versa). **(ii)**: comparison of the distance of small tumor cells and blood cells from the inner wall at different flow rates of the 2nd sheath.

Due to the inherent differences between deformable cells and rigid particles, the experimental conditions need to be re-optimized with tumor cell lines. In the first-stage spiral microchannel, the cell motility was consistent with the results of fluorescent microspheres (Figure 4A). Therefore, the flow rate of the 2nd sheath should be optimized. We measured the average diameter of the three kinds of tumor cell lines (Supplementary Figure S8C). In this study, 15.0-μm tumor cell was considered as the average standard diameter, and 12.5-μm tumor cell was regarded as the typical smaller diameter. The deflection effect of the 2nd sheath at the 1st bifurcation was quantified by capturing the cell trajectories. Deflection displacement distance was chosen as the main observation index. The results showed that the deflection displacement distance of 12.5-μm tumor cell was always greater than that of 15-μm tumor cell no matter what magnitude the 2nd sheath flow rate was (Figure 4B). Comparing the degree of deflection of blood and tumor cells, the deflection distance of both types of cells gradually increased with the accelerated flow rate of 2nd sheath flow rate (Figures 4A,B). The deflection distance of the blood cell flow trajectory was smaller at the 2nd sheath flow rate of 500 μl min^−1^. Part of blood cells entering the second-stage spiral microchannel reduced the sorting efficacy and purity of tumor cells. However, when the 2nd sheath flow rate was adjusted to 700 μl min^−1^, some smaller tumor cells were deflected by the fluid into the 1st waste channel, resulting in the loss of some tumor cells. Therefore, the 2nd sheath flow rate of 600 μl min^−1^ was selected as the optimal value. Under the optimum condition (sample:1st sheath:2nd sheath = 300:900:600 μl min^−1^), we captured the isolated tumor cells via the high-speed camera (Supplementary Video S2). Overall, the channel dimensions optimized on the basis of particle results should work well with an actual cell sample; however, the optimal operational flow rate would be slightly different, as the interaction between fluid and deformable cells introduces additional lift force. This additional force affects the exact equilibrium position of cells within the channel cross-section (Warkiani et al., 2016), which also explains that smaller microspheres should have a greater degree of deflection than larger ones, while in practice, at the same flow rate, 16.5 μm microspheres (Figure 3C) deflected more than 15.0-μm tumor cell (Figure 4A).

**FIGURE 5:**
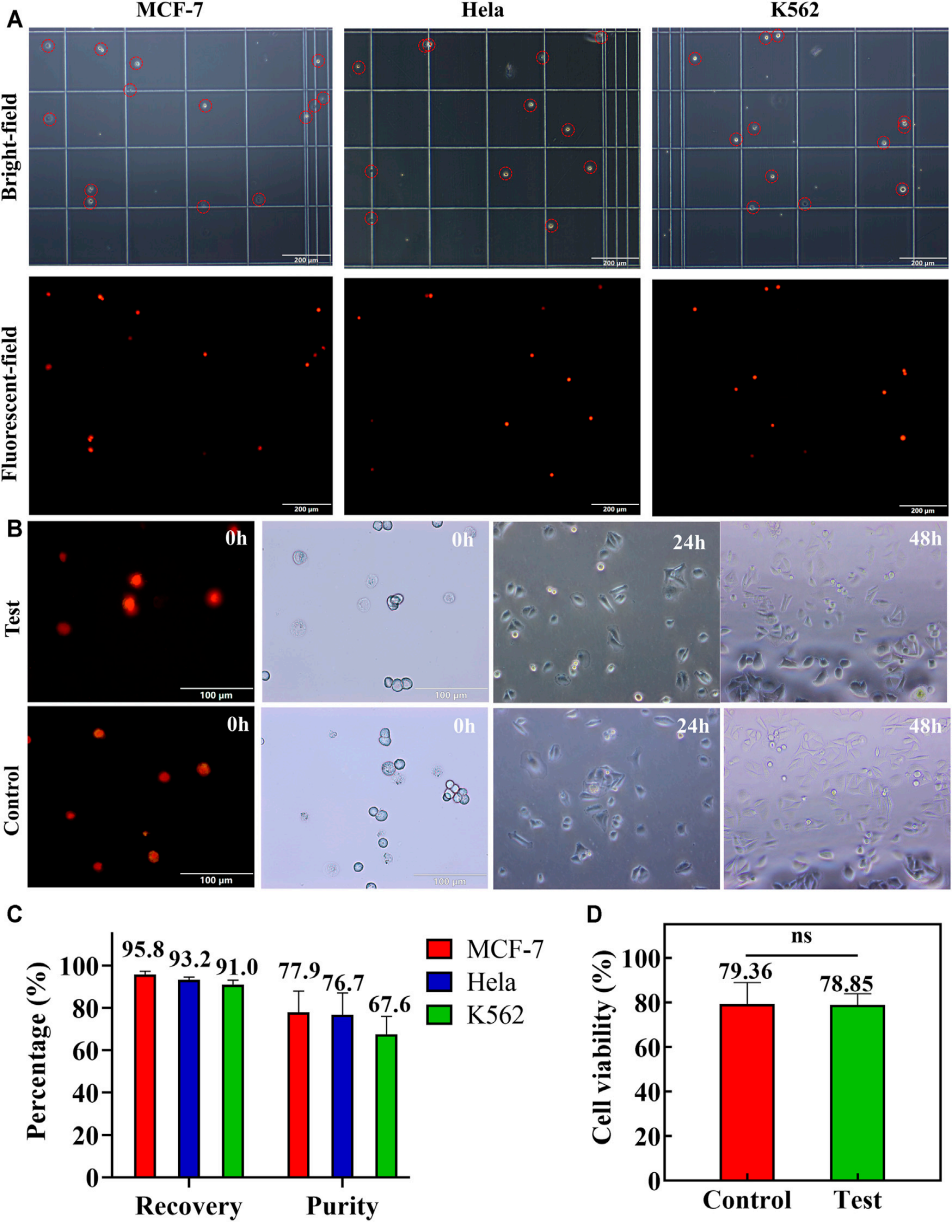
Spiked MCF-7, HeLa, and K562 cells were applied to test the double spiral microchannel. **(A)** Microscopic brightfield (red circle marked tumor cells) and fluorescence images of MCF-7, HeLa, and K562 collected from the target outlet. The scale bar is 200 μm. **(B)** Short-and long-term cell activity tests. Colored cells are apoptotic cells or dead cells. The scale bar is 100 μm. **(C)** Recovery and purity of three cells in the target outlet. **(D)** Comparison of the short-term cell viability between the test and control groups. Ns: *p* > 0.05.

Furthermore, the enrichment efficiency of the double spiral microchannel was characterized. Although double sheath fluids were brought into the double spiral microchannel, the collected volume in the target outlet was equal to the initial volume of sample (Supplementary Figure S9). When the number of spiked CTCs was less than 10^4^ cells ml^−1^, appropriate concentration and additional enrichment steps were beneficial for enumerating rare cells. The flow rates of sample, 1st sheath, and 2nd sheath were, respectively, adjusted to 1,600, 100, and 100 μl min^−1^, and the final collected volume would be condensed about 8 times. If a higher concentration of tumor cells is required, a secondary enrichment step is necessary. The tumor cells recovered from the target outlet were enumerated and analyzed (Figure 5A). There were high cell density and purity of tumor cells with only minimal blood cell interference. The short-term cell activity test showed that some cells were induced to undergo early apoptosis, but the difference in cell activity between the test and control groups was not significant (Figure 5D). The collected cells were cultured directly, and their cell viability and morphology were consistent with those of the control group (Figure 5B). The recovery rate of the three types of cells collected exceeded 91%, and the sorting purity averaged 74% (Figure 5C). Compared to other passive methods for sorting CTC chips (Supplementary Table S2), samples pumped into the double spiral microchannel did not require lysis of RBCs or high-fold dilution pretreatment and also successfully obtained cells with relatively high activity and purity. Therefore, sorting of high-purity and high-activity rare cells via double spiral microchannel has promising practical applications in clinical trials.

### 4.4 Application of Clinical Samples

**FIGURE 6:**
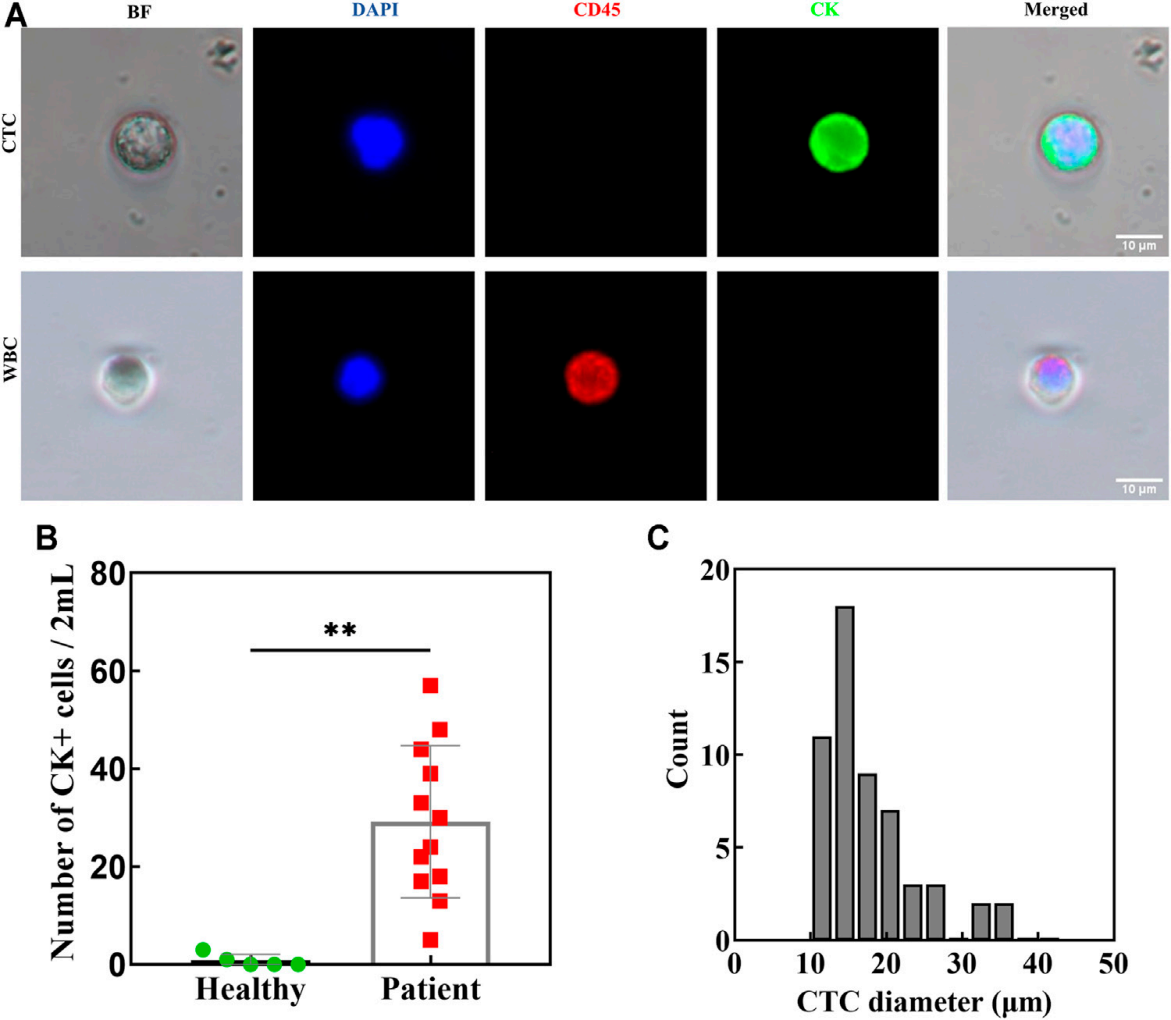
Blood from patients with lung/breast cancer (*n* = 12) and healthy donors (*n* = 5) was processed using a double spiral chip. **(A)** Immunofluorescence staining of isolated cells. **(B)** CK positive cells in both patients and healthy as indicated. **p < 0.05. **(C)** The diameter and the corresponding number of isolated CTCs in patients.

To validate the clinical efficacy of our double spiral microchannel, we selected healthy adults (*n* = 5) as a negative control group and cancer patients (*n* = 12) as an experimental group (Supplementary Table S3). As a matter of priority, whole blood (∼2 ml) with HCT of ∼40% was diluted about 10 times (∼20 ml). Our chip could complete the sorting process in approximately 1 h. During the separation, the trajectories of CTCs were captured by high-speed CCD (Supplementary Video S3). After flowing through the first-stage spiral microchannel, CTCs moved into the second-stage spiral microchannel, and they were collected from the target outlet finally. The presence of isolated CTCs was determined by immunostaining with DAPI (DNA), PE-CD45 antibodies, and FITC-cytokeratin (CK) antibodies (Figure 6A). DAPI^+^-CD45^−^-CK^+^ cells were scored as CTCs. The CTCs were detected in 12 out of 12 patient samples with counts ranging from 5 to 57 CTCs per 2 ml of blood, which verified the high sensitivity of the double spiral microchannel for detecting CTCs.

A larger number of CTCs detected from peripheral blood of patients often indicate a higher degree of malignancy of tumor, especially for bone metastasis (Cheng et al., 2014; Nel et al., 2014). As our research showed, the number of CTCs in patients with bone metastasis was relatively numerous (Supplementary Table S3). Cytokeratin-positive epithelial cells were also detected in healthy volunteers (0–3 per 2 ml), but a distinct detection threshold was seen versus the patient samples (Figure 6B). There is a baseline value predicting metastatic disease between healthy and cancer patients, which is consistent with previous reports in the literature. (Krebs et al., 2011). Relatively accurate critical value requires further investigation with more samples. Importantly, the presence of CTCs above baseline has been very convincingly shown to be predictive of a poor prognosis in patients with various cancer types and in large clinical trials (Pantel and Alix-Panabières, 2019). We also evaluated the size and shape of isolated CTCs (Figure 6C). They ranged from 10.8 to 34 μm, with an average diameter of ∼17.5 μm. The minimum diameter of the isolated CTCs did not coincide with the theoretical calculation, which may be caused by the adhesion behavior between the cells or cell clusters. Intact and active CTCs are of great value for clinical applications, such as capturing high-purity CTCs for sequencing to assist in clinical diagnosis and medication guidance. As the number of CTCs in the clinical sample was unpredictable, the sorting purity of samples with a large number of CTCs was higher than that with a small number of CTCs. However, the purity of CTCs isolated from clinical samples was not as high as that of spiked CTCs. One reason for this result was that the spiked CTCs were relatively high activity and large size cells obtained by *in vitro* culture and centrifugal screening, which was beneficial for raising the efficiency and purity of sorting. Another reason was that the CTC identification method by immunofluorescence staining could not avoid the limitation of immunological heterogeneity. Thus, we will perform a thorough characterization of the isolated CTCs by electrical impedance or molecular diagnostic techniques and conduct clinical verification of the therapeutic efficacy and prognosis of cancer in the next step.

## 5 Conclusion

In this study, we designed a novel double spiral microchannel, combining the double sheath fluids with a two-stage spiral separation system, which efficiently enhanced the separation and enrichment of rare cells. The separation mechanism is size-based inertial focusing and particle deflection theories. First, the fluid was simulated under different flow conditions to determine the structural parameters of microchannel and to further understand the working principle of the chip. Second, different microspheres were employed to characterize the properties of inertial focusing and particle deflection. The optimum flow rate was determined for the separation of the binary microsphere mixture, and the performances of single and double spiral microchannels were compared. Subsequently, we studied the effects of the hematocrit and the 2nd sheath on the performance of the double spiral microchannel separation using the spiked tumor cell lines. An only 10-fold dilution of whole blood was required to achieve tumor cell recovery of 91% and separation purity of 74%. Finally, we validated the applicability of the double spiral microchannel using the clinical cancer blood samples. Rare CTCs were successfully isolated from all patients’ blood samples. Compared with existing techniques for inertial focusing and sorting of CTCs, simple sample pretreatment, low sample loss, high separation efficiency, and high purity are unique advantages for this chip. We expect that our research will inspire more researchers and stimulate the design and development of new microchannels. At the same time, our research showed that it was clinically convenient and feasible to separate CTCs in blood samples via a double spiral microchannel. In the future, the combination of downstream detection and identification analysis (i.e., electrical impedance or molecular biology) of double spiral microchannel could have great potential for the early diagnosis and prognosis of cancer.

## Data Availability

The original contributions presented in the study are included in the article/Supplementary Material, and further inquiries can be directed to the corresponding author.

## Supplementary Material

Supplementary Video 1 The 10.4 and 16.5 μm fluorescent microspheres flowing through the first bifurcation and second bifurcation of the double spiral channel at a 1.2 ml min^−1^ flow rate. The 16.5 μm microspheres flowed through the first- and second-stage spiral microchannels and finally collected from the target outlet, while a large percentage of 10.4 μm microspheres flowed out from 1st waste and a few proportions of them from 2nd waste.

Supplementary Video 2 Spiked MCF-7 cells into 10-fold dilution blood flowing in double spiral microchannel. The video was captured at the first and second bifurcations.

Supplementary Video 3 obviously isolated the CTCs from the peripheral blood of cancer patients.
