# Supplementary material for: Enhanced Separation Efficiency and Purity of Circulating Tumor Cells Based on the Combined Effects of Double Sheath Fluids and Inertial Focusing
Source: Front Bioeng Biotechnol. 2021 Oct 27;9:750444. doi: 10.3389/fbioe.2021.750444 (PMC8578950; doi:10.3389/fbioe.2021.750444)
Supplement: Supplementary file 4 [file datasheet1.docx]

Supplementary Material

Enhanced separation efficiency and purity of circulating tumor cells based on the combined effects of double sheath fluids and inertial focusing

Bo-Wen Li^1^^†^, KunWei^1†^, Qi-Qi Liu^2^, Xian-Ge Sun^1^ Ning Su^1^, Wen-Man Li^1^, Mei-Yun Shang^1^, Jin-Mi Li^1^, Dan Liao^1^, Jin Li^1^, Wei-Ping Lu^1^, Shao-Li Deng^1^, Qing Huang^1^*

^1^Department of Laboratory Medicine, Daping Hospital, Army Medical University, Chongqing 400042, China;

^2^Department of Nursing, Children’s Hospital of Chongqing Medical University, Chongqing 400015, China;

^†^These authors share first authorship.

*Correspondence:

Qing Huang
[qinghuang@tmmu.edu.cn](mailto:qinghuang@tmmu.edu.cn)

**Document 1.** Formulas for separation efficiency, purity, recovery, and enrichment ratio.

The separation efficiency of rare cells was calculated using the following equation:

Separation efficiency = $\frac{\boldsymbol{N}_{\boldsymbol{(target outlet)}}}{\boldsymbol{N}_{\boldsymbol{(1}\boldsymbol{st waste +2}\boldsymbol{nd waste+target outlet)}}}$ × 100% (1)

where N _(target outlet)_ is number of target cells in target outlet, and N _(outlet 1+outlet 2+target outlet)_ is numbers of target cells in total outlets.

The purity was calculated using the following equation:

Purity = $\frac{\mathbf{N}_{\mathbf{(target outlet)}}}{\boldsymbol{T}_{\mathbf{(}\boldsymbol{target} \boldsymbol{outlet}\mathbf{)}}}$× 100% (2)

where T _(_*_target outlet_*_)_ is the number density of total cells collected from the target outlet.

The recovery of rare cells was calculated using the following equation:

Recovery = $\frac{\boldsymbol{N}_{\boldsymbol{(target outlet)}}}{\boldsymbol{N}_{\boldsymbol{(inlet)}}}$ × 100% (3)

where N _(inlet)_ is the number density of target cells injected into the sample inlet.

The enrichment ratio of rare cells was calculated using the following equation:

Enrichment ratio = $\frac{\boldsymbol{R}_{(\boldsymbol{target} \boldsymbol{outlet})}}{\boldsymbol{R}_{(\boldsymbol{inlet})}}$ (4)

where R _(target outlet)_ is the ratio of cancer cells to blood cells in the target outlet, and R _(inlet)_ is ratio of cancer cells to blood cells in the sample inlet.

**
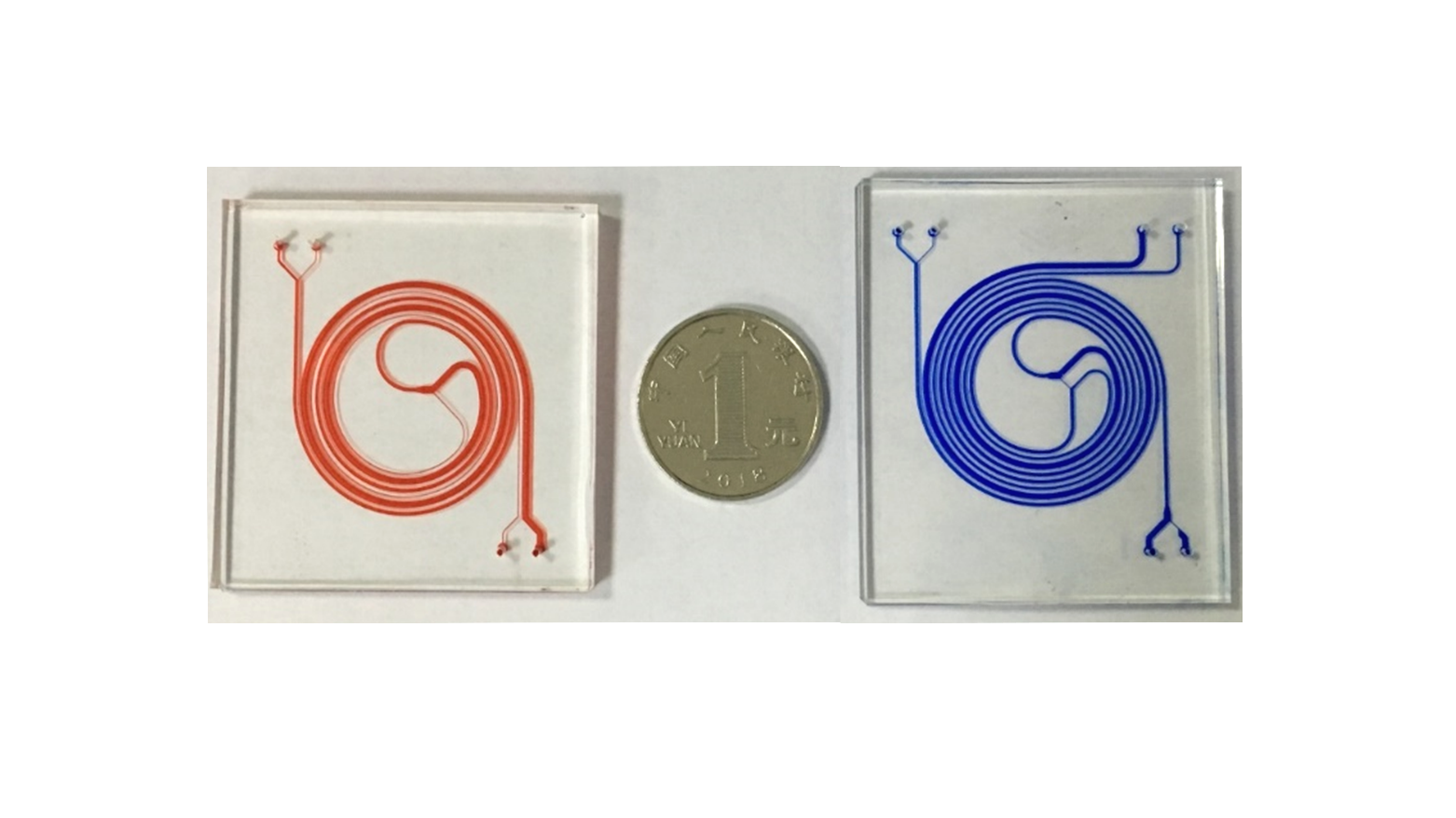
**

**Fig. S1.** Photograph of both single (left) and double (right) spiral microchannels used in this study..

**
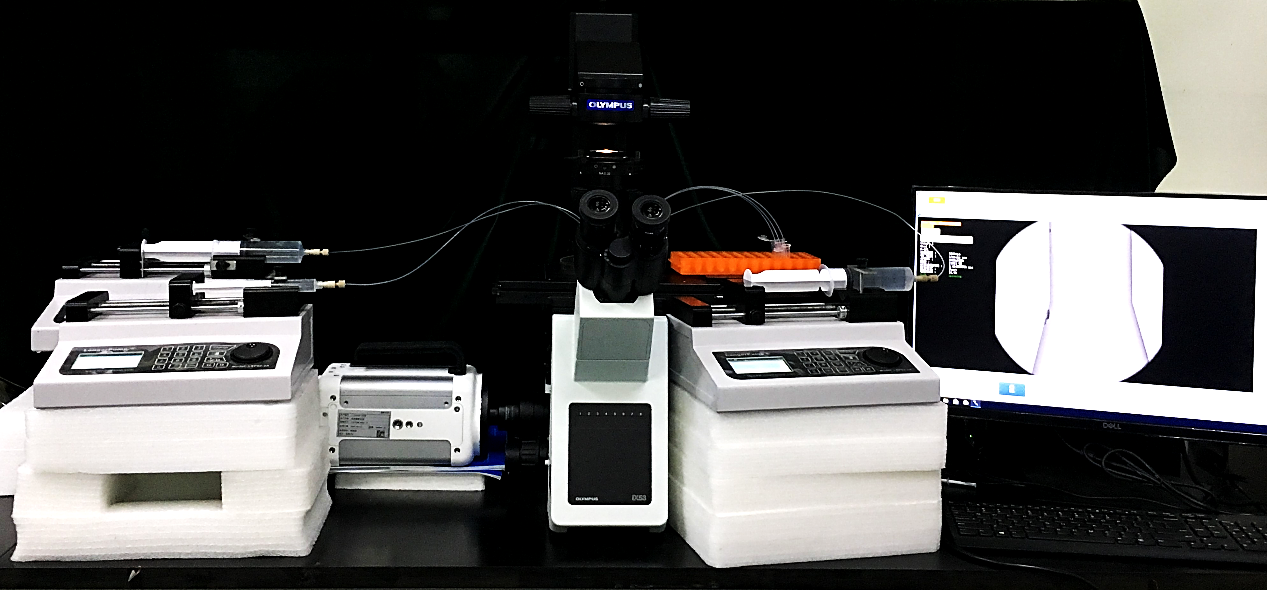
**

**Fig. S2.** Experimental platform system. The system consists of a double spiral chip, a microscopic system (including microscope, CCD camera and computer) for imaging and three syringe pumps for injecting sample and sheaths.

**
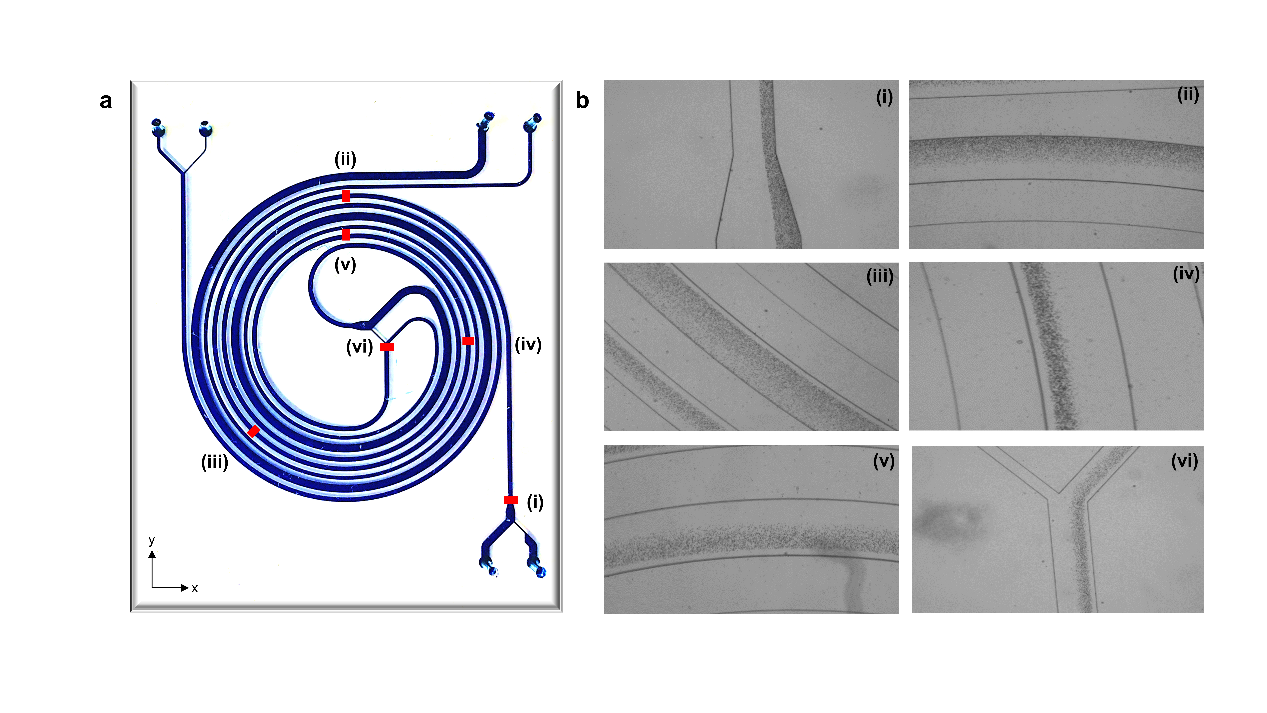
Fig. S3.** Photos of the trajectory of the diluted whole blood at different positions in the double spiral channel. Blood cells followed Dean’s secondary flow theory and flowed regularly with a back-and-forth motion between the inner and outer wall.

**
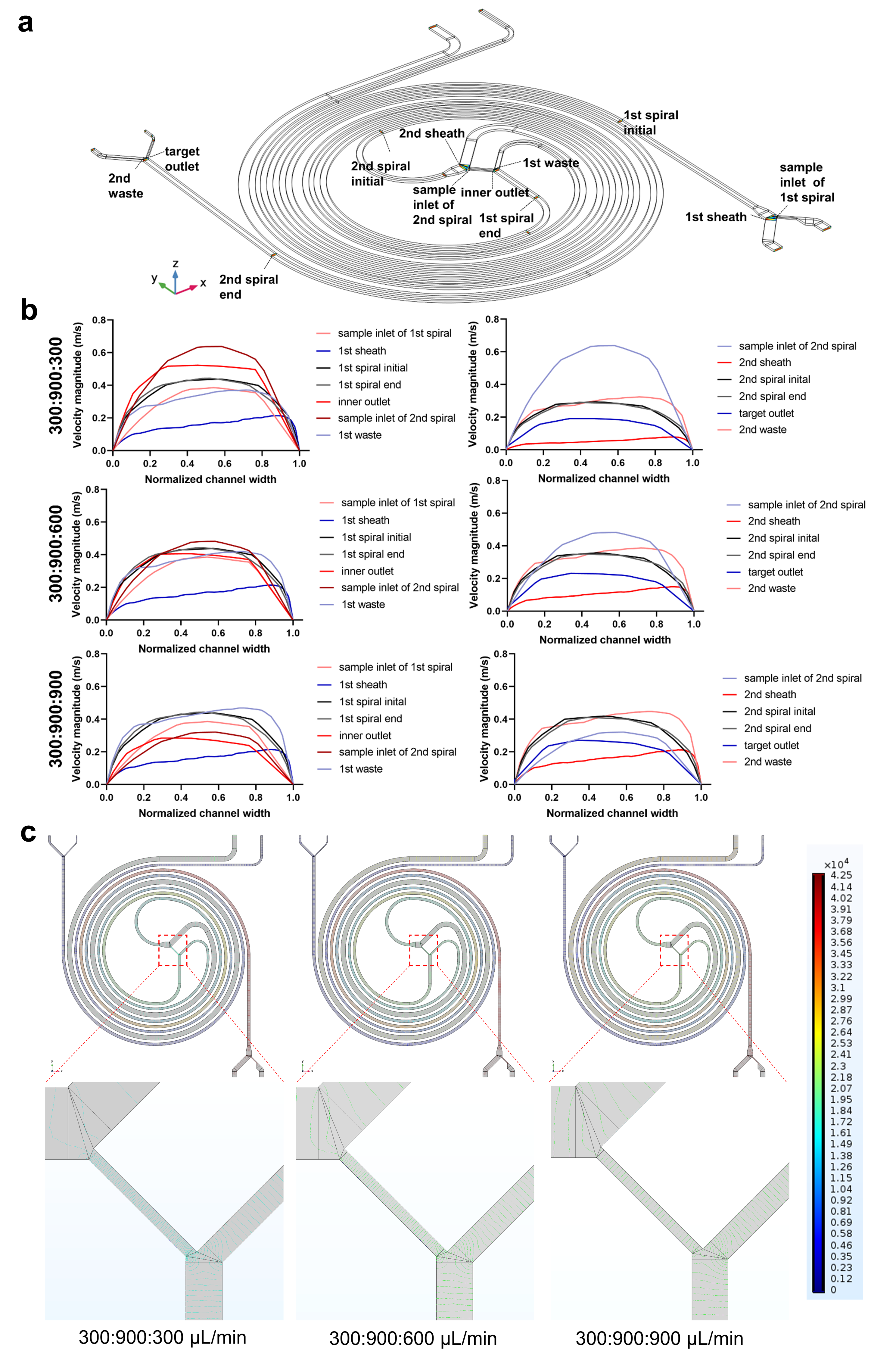
**

**Fig. S4.** Flow rate and pressure simulation conducted by COMSOL Multiphysics. (a) The image shows the overall geometry of microchannels and the name definition of the different cross-sections. (b) Contrast curve of velocity distribution of the different 2nd sheath at different positions. The 0.0 coordinate of the X axis represents the inner wall, and 1.0 represents the outer wall. (c) Simulation diagram of chip channel pressure at different flow rates.

**
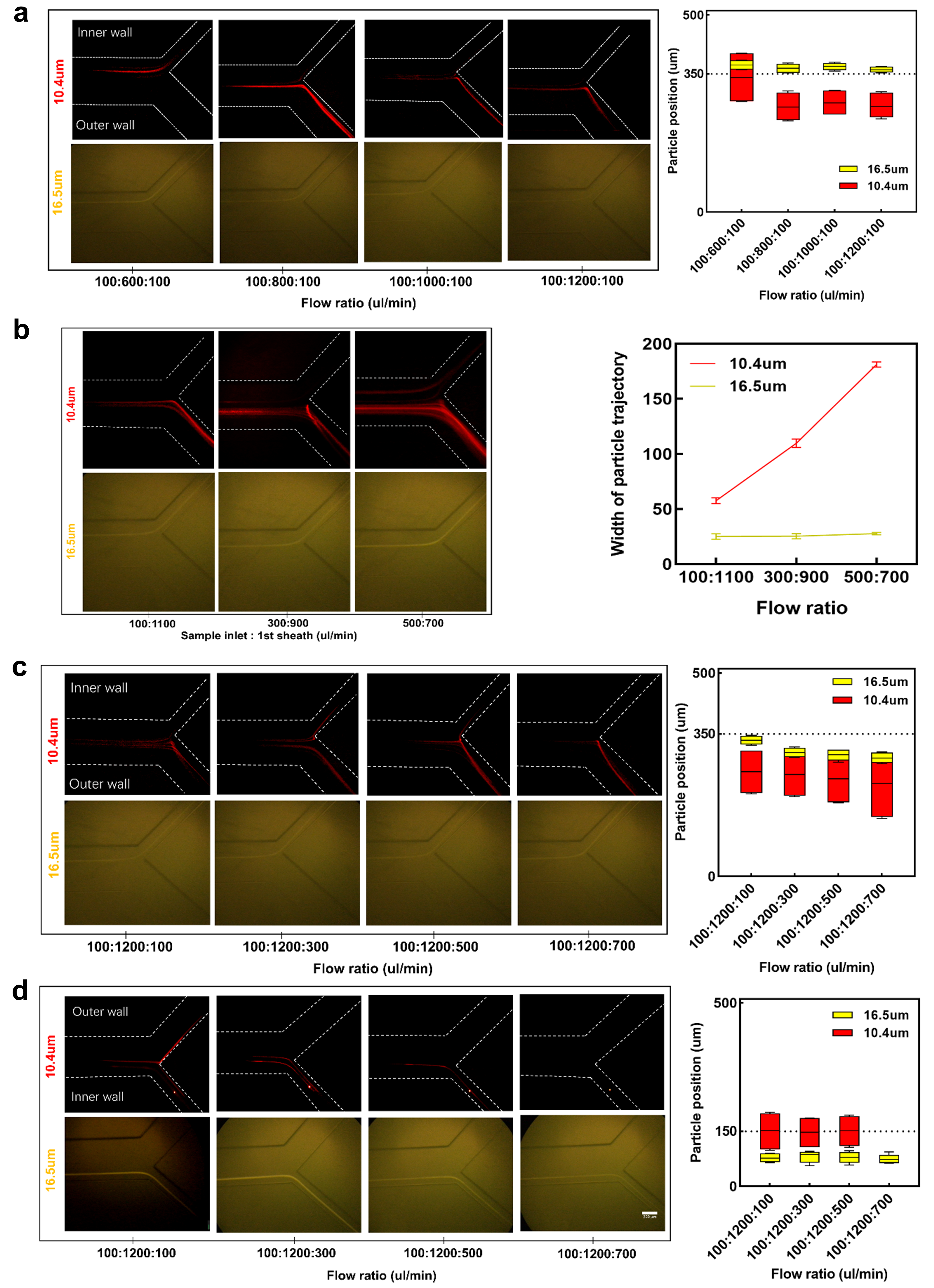
**

**Fig. S5.** Images illustrated the trajectories of the 16.5 and 10.4 μm fluorescent microspheres at different flow rates. The lateral positions of the 16.5 and 10.4 μm microspheres are displayed in the extraction channel with a 500 μm width at various flow rates of sample, 1st and 2nd sheath. The scale bar is 200 μm. (a) It showed the effect of different flow rate of the 1st sheath on particle flow. (b) The image showed the relationship between flow rate ratio and the width of two types of particle trajectories. (c) The effect of the 2nd sheath flow rate on the particle distribution positions at the first and (d) second bifurcation.


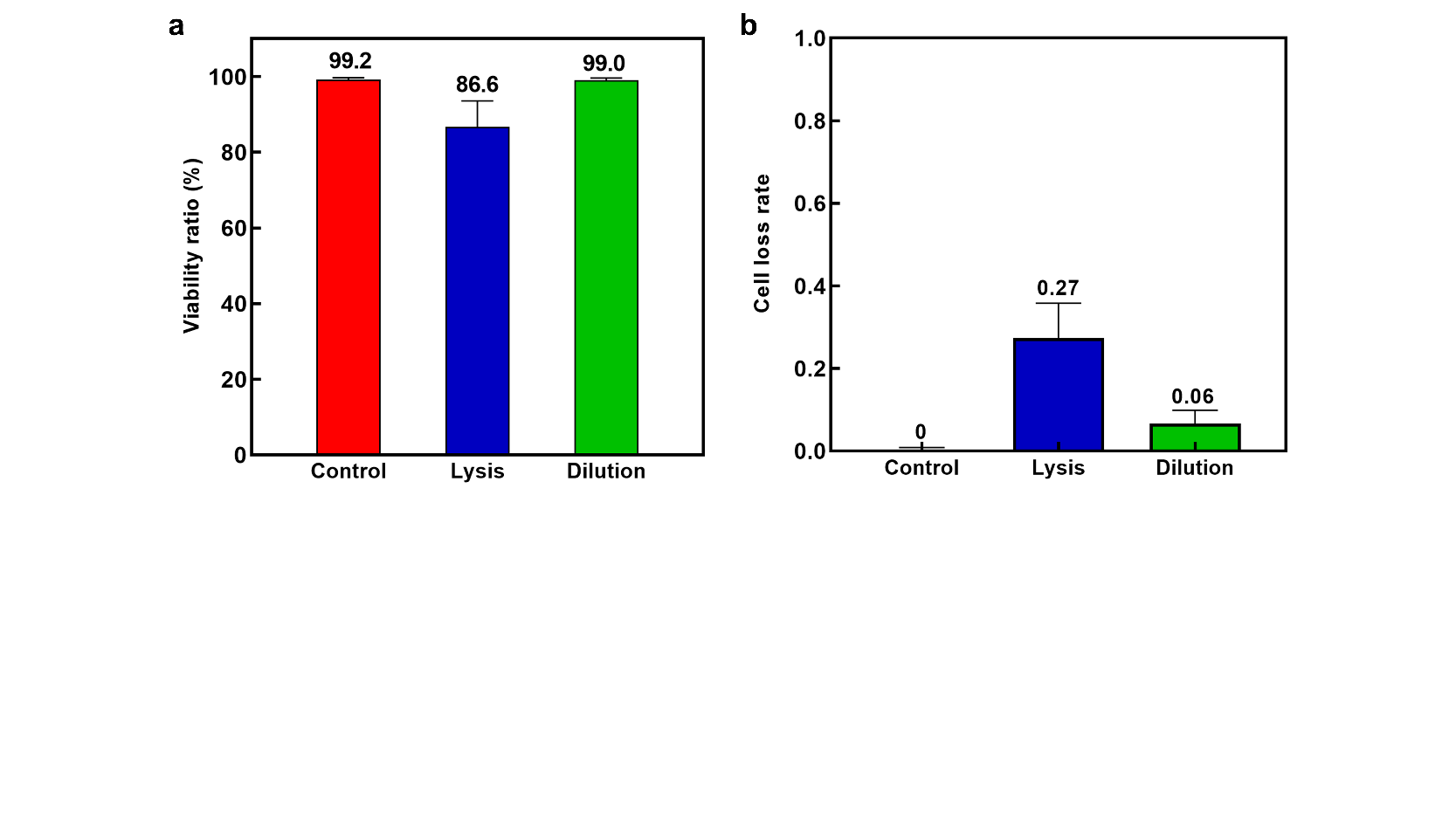


**Fig. S6.** (a). Effect of lysis and dilution on cell viability. (b). Cell loss rate during lysis and dilution treatment. Data are presented as mean$\pm s.d.$ (n=3).

**
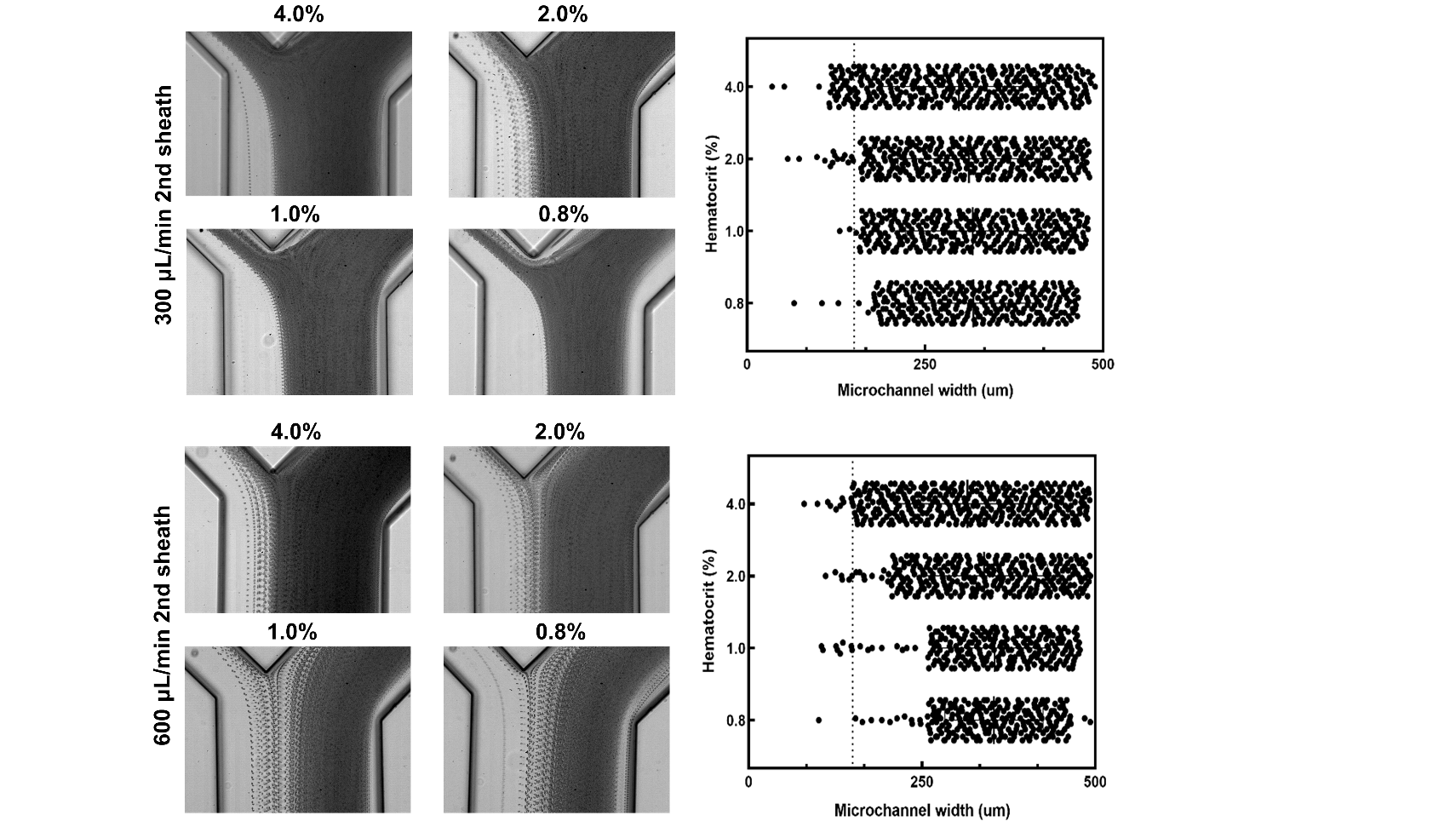
**

**Fig. S7**. Left: high speed camera (20000 fps) captured the image of the cell movement trajectory in different dilutions of whole blood (4%, 2%, 1%, and 0.8%). The images are superimposed by more than 3000 photos. Right: the width of the background cell flow distribution under different dilution factors. The dotted line represents 150 μm of the first bifurcation microchannel.


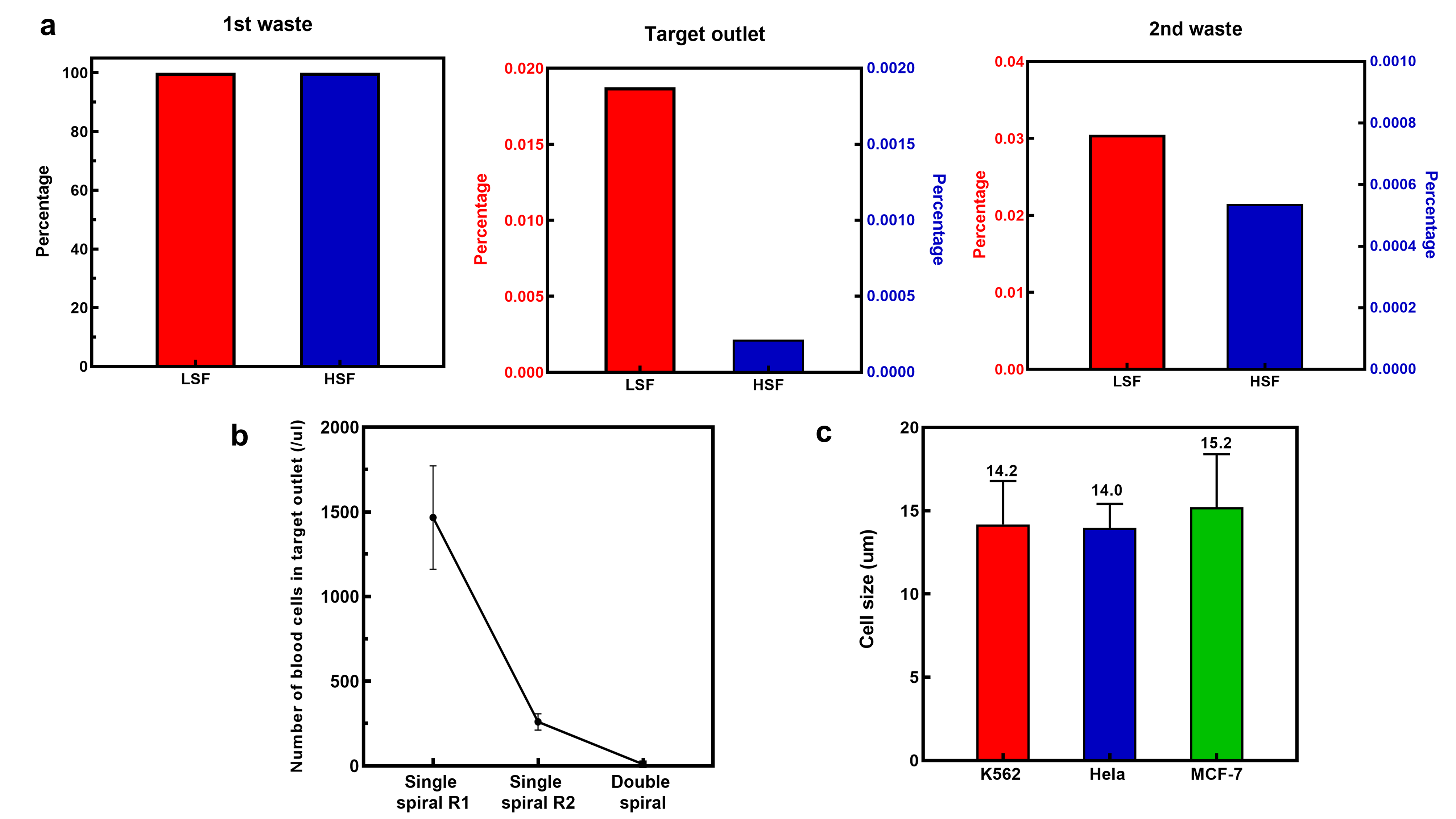


**Fig. S8.** (a) Effect of low sheath fluid 2 (LSF) and high sheath fluid 2 (HSF) on blood cells distribution at different outlets. (b) A comparison of blood cells with single spiral secondary separation and double spiral in target outlet. (c) The diameters of different tumor cells were measured. Data are presented as mean$\pm s.d.$ (n>55).

**
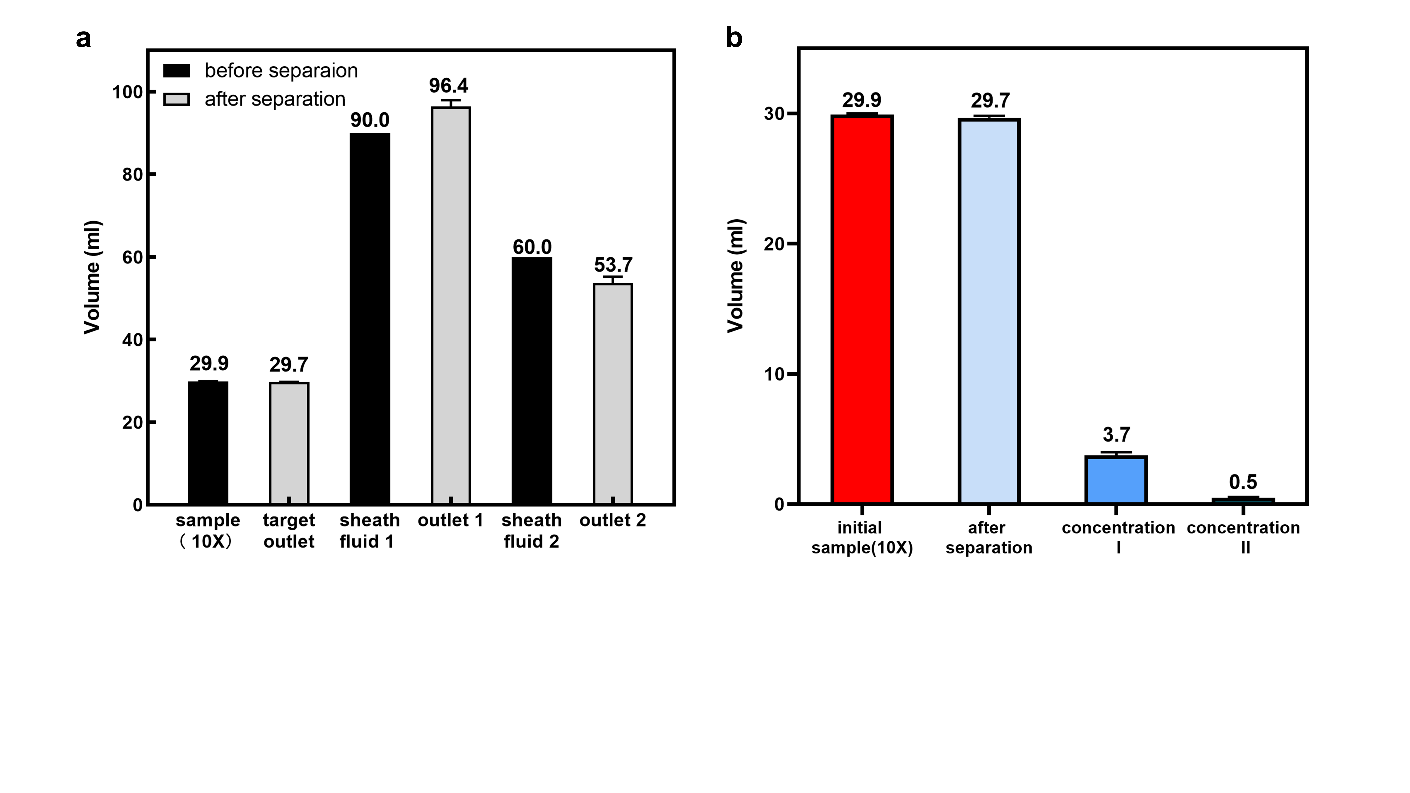
**

**Fig. S9.** (a) Comparison of volume at different inlets and outlets (n=3). (b) The volume change of the initial sample volume after separation and concentration I, II. Data are presented as mean$\pm s.d.$ (n=3).

**Table S1.** Optimized and detailed parameters of the double spiral chip.

| **Name** | **Value** | **Unit** |
| --- | --- | --- |
| External dimension | 50*60 | mm |
| Number of spiral channels | 3.5 | loops |
| Turns of one spiral channel | 1.75 | loops |
| Channel spacing | 500 | μm |
| Width of sheath fluid channels | 850 | μm |
| Width of outlet 1 | 350-395 | μm |
| Width of outlet 2 | 350 | μm |
| Angle of bifurcations | 90° | - |
| Initial radius | 0.4 | cm |


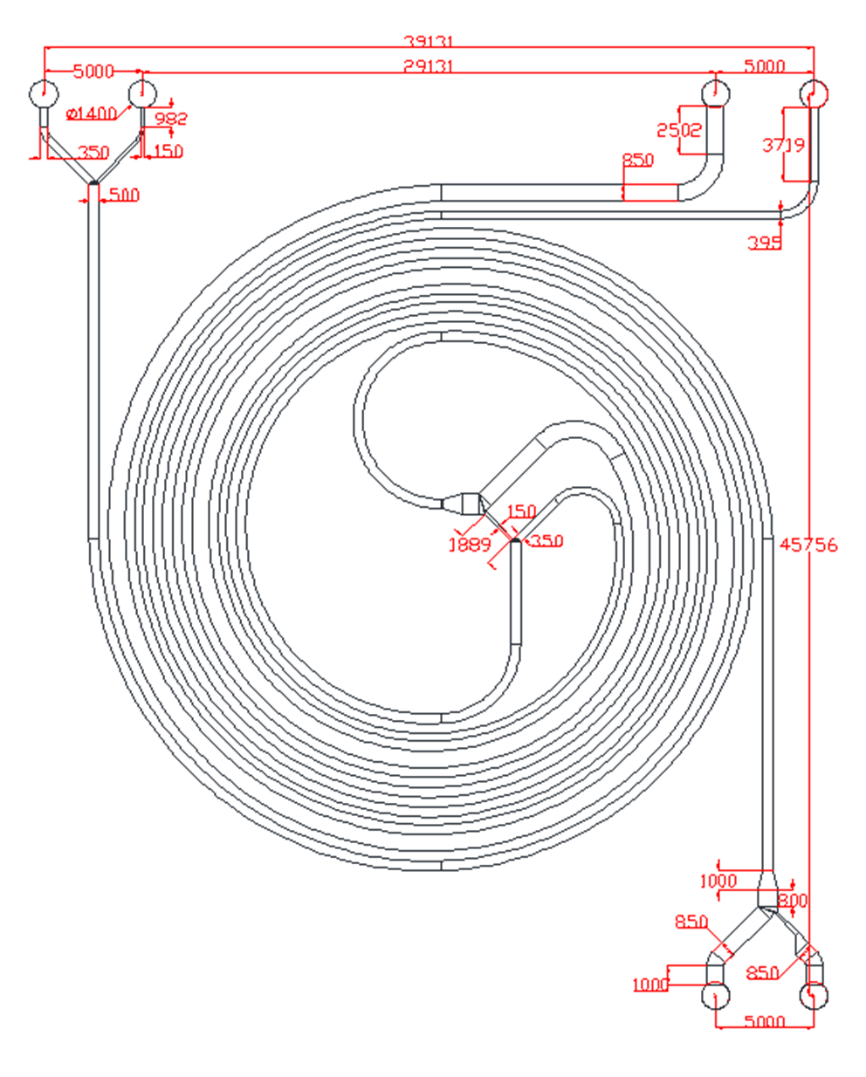


**Fig. S10.** More detailed dimensional parameters are marked at the side of the double spiral microchannel structure. (All of units are micron)

**Table S2.** Comparison of the characteristics and performance of previously reported and our designed spiral microchannels.

| The number of spiked CTCs | Through-put | Cell viability | Pretreatment | Purity | Features | Reference number |
| --- | --- | --- | --- | --- | --- | --- |
| MCF-7  (~10^5^/mL) | 100 μL/min | > 98% | ~2×  dilution (20% hematocrit) | CTCs/leukocytes ~10% | Catheter connect two single spiral | Hou,  et, al.[1] |
| MCF-7  (~200-400/mL) | 550 μL/min | > 90% | Sedimentation and fractionation RBC | Not given (97.91% leukocyte depletion) | Two consecutive single spiral | Kim,  et, al.[2] |
| MCF-7  (10^4^/mL) Hela  (10^2^/mL) | 333 μL/min | Intact spherical shape | 50×  dilution | <1% | Double spiral | Sun,  et, al.[3] |
| MCF-7 (500/mL) | 400 μL/min | >95% | ~20×  diluted (∼5 × 10^7^/mL)  Lysis RBC (10^6^/mL) | 17.68%  15.48% | Single spiral, deterministic lateral displacement | Xiang,  et, al.[4] |
| MCF-7, Hela, A549 | 750 μL/min | Excellent viabilities | 100×  dilution | 29.2% | Contraction/ expansion single spiral | Gou,  et, al.[5] |
| MCF-7, A549  (500/mL) | 1200 μL/min | > 95% | ~20×  dilution (∼5 × 10^7^/mL) | 51.47%-93.60% (higher dilution) | Single spiral, serpentine, magnetic sorter | Huang,  et, al.[6] |
| K562, Hela, MCF-7 (10^2^-10^3^/mL) | 300 μL/min | 99%  (Relative value) | ~10×  dilution (4% hematocrit) | 74% (>99.999% blood depletion) | Double spiral | Our work |

[1] H.W. Hou, M.E. Warkiani, B.L. Khoo, Z.R. Li, R.A. Soo, D.S.W. Tan, W.T. Lim, J. Han, A.A.S. Bhagat, C.T. Lim, Isolation and retrieval of circulating tumor cells using centrifugal forces, Sci. Rep. 3 (2013).<http://dx.doi.org/10.1038/srep01259>

[2] T.H. Kim, H.J. Yoon, P. Stella, S. Nagrath, Cascaded spiral microfluidic device for deterministic and high purity continuous separation of circulating tumor cells, Biomicrofluidics 8(6) (2014).<http://dx.doi.org/10.1063/1.4903501>

[3] J.S. Sun, M.M. Li, C. Liu, Y. Zhang, D.B. Liu, W.W. Liu, G.Q. Hu, X.Y. Jiang, Double spiral microchannel for label-free tumor cell separation and enrichment, Lab Chip 12(20) (2012) 3952-3960.<http://dx.doi.org/10.1039/c2lc40679a>

[4] N. Xiang, J. Wang, Q. Li, Y. Han, D. Huang, Z. Ni, Precise Size-Based Cell Separation via the Coupling of Inertial Microfluidics and Deterministic Lateral Displacement, Anal. Chem. 91(15) (2019) 10328-10334.<http://dx.doi.org/10.1021/acs.analchem.9b02863>

[5] Y.X. Gou, S. Zhang, C.K. Sun, P. Wang, Z. You, Y. Yalikun, Y. Tanaka, D.H. Ren, Sheathless Inertial Focusing Chip Combining a Spiral Channel with Periodic Expansion Structures for Efficient and Stable Particle Sorting, Anal. Chem. 92(2) (2020) 1833-1841.<http://dx.doi.org/10.1021/acs.analchem.9b03692>

[6] D. Huang, N. Xiang, Rapid and precise tumor cell separation using the combination of size-dependent inertial and size-independent magnetic methods, Lab Chip 21(7) (2021) 1409-1417.<http://dx.doi.org/10.1039/d0lc01223h>

**Table S3.** Patient demographic information for samples used for enumeration of CTCs.

| Patient | Gender | Age | Cancer Type | Tumor Histology | Stage | Tumor Spread | | CTC count /2mL |
| --- | --- | --- | --- | --- | --- | --- | --- | --- |
| 1 | Female | 64 | Lung | ADC | IV | Lymphatic | 44 | |
| 2 | Female | 50 | Breast | ILC | II | Lung | 24 | |
| 3 | Female | 57 | Lung? | - | - | - | 13 | |
| 4 | Male | 69 | Lung | ADC | IV A | Lymphatic | 33 | |
| 5 | Female | 63 | Lung | ADC | III | Lymphatic | 30 | |
| 6 | Male | 46 | Lung | ADC | IV | Bone | 48 | |
| 7 | Female | 62 | Lung | ADC | IV | Brain, Bone | 57 | |
| 8 | Male | 53 | Lung | SCC | III A | Lymphatic | 17 | |
| 9 | Male | 68 | Lung | ADC | IV | Lymphatic | 5 | |
| 10 | Male | 69 | Lung | SCLC | III B | Lymphatic | 18 | |
| 11 | Male | 54 | Lung | SCC | - | - | 39 | |
| 12 | Male | 65 | Lung | ADC | - | - | 22 | |
| 13 | Female | 67 | - | - | - | - | 1 | |
| 14 | Female | 28 | - | - | - | - | 0 | |
| 15 | Male | 50 | - | - | - | - | 0 | |
| 16 | Male | 68 | - | - | - | - | 3 | |
| 17 | Female | 19 | - | - | - | - | 0 | |

Note: Patient 3 had not further examination after the initial examination; patients 11 and 12 were initially diagnosed with cancer; 13-17 patients were healthy donors.
